# The efficacy and safety of acupuncture treatment for peripheral facial paralysis: an overview of systematic review and meta-analysis

**DOI:** 10.3389/fneur.2025.1669551

**Published:** 2025-11-11

**Authors:** ZhiLin Huang, Yating Zhang, BIxiang Zha, Ping Wang, Song Li, Ziyu Ye, Sichen Liu, Linying Wang, Jun Yang

**Affiliations:** 1 The First Clinical Medical College of Anhui University of Chinese Medicine, Hefei, China; 2 The First Affiliated Hospital of Anhui University of Chinese Medicine, Hefei, China; 3 Heilongjiang University of Chinese Medicine, Harbin, China

**Keywords:** systematic review, acupuncture, peripheral facial paralysis, overview, meta-analysis

## Abstract

**Background:**

Many studies have investigated the efficacy and safety of acupuncture in the treatment of peripheral facial palsy (PFP), but the results have been inconsistent. This study aimed to evaluate systematic reviews (SRs) and meta-analyses (MAs) of the treatment of PFP by integrating acupuncture, providing a basis for clinical treatment.

**Methods:**

We searched Wanfang, VIP, CNKI, CBM, Web of Science, PubMed, and Cochrane Library databases for SRs and MAs related to acupuncture treatment for PFP, from the establishment of the databases to May 1, 2025. We evaluated the methodology, reporting quality, and evidence quality of the included studies using the AMSTAR2, PRISMA, and GRADE tools.

**Results:**

This study included a total of 17 SRs and MAs. The AMSTAR2 assessment results showed that three studies were rated as having low methodological quality, while 14 studies were considered to have very low methodological quality. The PRISMA results indicated that two studies were of high quality, nine were of moderate quality, and six were of low quality. GRADE results indicated that only two items provided moderate-quality evidence, 22 items provided low-quality evidence, and 13 items provided very low-quality evidence. Acupuncture can improve the clinical efficacy of PFP, reduce recovery time, and has few adverse reactions.

**Conclusion:**

Acupuncture is effective in treating PFP, but methodological shortcomings in existing studies have resulted in limited evidence. In the future, it is necessary to follow the principles of evidence-based medicine to improve the quality of relevant RCTs and SRs and MAs studies and enhance the credibility of the evidence.

**Systematic review registration:**

PROSPERO, identifier: CRD420251033106.

## 1 Introduction

Peripheral facial palsy (PFP) is a facial motor dysfunction caused by lower motor neuron lesions, representing a prevalent clinical disorder of the peripheral nervous system (1). The global incidence rate is estimated to range between 15 and 23 cases per 100,000 individuals annually, with the associated treatment costs imposing a substantial economic burden on both patients and society at large (2). Clinically, this condition presents as unilateral facial muscle weakness or paralysis, typified by effacement of forehead wrinkles on the affected side, incomplete palpebral closure, impaired buccal inflation, and contralateral deviation of the oral commissure (3). Furthermore, a subset of patients may experience hypogeusia or hypoacusis, accompanied by retroauricular pain (4). Although PFP is a self-limiting disease, a significant number of patients still experience varying degrees of sequelae, causing them emotional distress and disrupting their lives (5, 6). Current therapeutic strategies for PFP encompass pharmacological interventions (e.g., corticosteroids and antiviral agents), surgical procedures, and alternative therapies, including acupuncture (7).

Acupuncture is a common non-pharmacological treatment for PFP, and its efficacy has been confirmed by several studies (8–10). PFP is also one of the 64 indications for acupuncture recognized by the World Health Organization (WHO) (11). Acupuncture demonstrates therapeutic efficacy in ameliorating PFP through the augmentation of functional connectivity between motor and sensory cortices, modulation of neuroplasticity, attenuation of inflammatory responses, enhancement of regional microcirculatory dynamics, and facilitation of multifaceted, multi-targeted neural structural and functional restoration (12–14). In recent years, the advancement of evidence-based medicine in Traditional Chinese Medicine (TCM) has led to the publication of numerous systematic reviews and meta-analyses (SRs/MAs) investigating the efficacy of acupuncture for PFP in both domestic and international journals, thereby offering preliminary empirical support for this therapeutic intervention. However, the validity of SR/MAs is inherently constrained by both the methodological quality of primary studies and researchers' proficiency in evidence synthesis techniques. Notable limitations include: (1) selection bias stemming from small sample sizes in included trials; (2) heterogeneity across acupuncture and moxibustion modalities; (3) variability in diagnostic criteria; and (4) inconsistency in outcome measures–all of which may compromise the reliability of derived conclusions. It is essential to integrate these SRs and MAs further, analyze the methodological shortcomings in existing studies, enhance the credibility of the evidence, and provide guidance for clinical decision-making (15, 16).

Therefore, this study aims to comprehensively summarize the methodological quality, reporting quality, evidence quality, and the results of a meta-analysis of SR/MA of acupuncture intervention for PFP using AMSTAR 2, the PRISMA statement, and the GRADE tool. The aim is to objectively evaluate the efficacy and safety of acupuncture in the treatment of PFP, providing a more reliable reference for clinical decision-making.

## 2 Materials and methods

### 2.1 Registration

This review was pre-registered on the PROSPERO platform on 15 April 2025 (registration number: CRD420251033106).

### 2.2 Inclusion and exclusion criteria

#### 2.2.1 Inclusion criteria

The inclusion criteria for this study are as follows: Eligible participants comprised individuals with a confirmed diagnosis of PFP, with no restrictions imposed regarding age, gender, geographical origin, disease phase, or clinical staging. The experimental group interventions predominantly consisted of acupuncture modalities (encompassing body acupuncture, electroacupuncture, scalp acupuncture, moxibustion, warm needle therapy, auricular acupuncture, and heat-sensitive moxibustion, among others, with exclusion of acupoint injection and needle-knife techniques). Control group interventions comprised all non-acupuncture therapeutic approaches, including conventional Western medicine regimens, traditional Chinese medicine treatments, placebo controls, and sham acupuncture. Primary outcome measures included the composite efficacy rate and complete remission rate, along with the facial paralysis motor function evaluation Scale, notably the House-Brackmann (H-B) grading system and the Facial Disability Index (FDI). Secondary outcomes encompassed electrophysiological parameters such as facial nerve conduction velocity (NCV). SRs and MAs about randomized controlled trials (RCTs) or controlled clinical trials (CCTs) investigating acupuncture for PFP were included.

#### 2.2.2 Exclusion criteria

The exclusion criteria for this study are as follows: Duplicate publications (including duplicate publications of Chinese literature in English journals) Published but withdrawn literature systematic reviews and Meta-analysis of basic research systematic reviews for which the full text was still unavailable after the authors were contacted Protocol for systematic reviews While both groups received acupuncture interventions, the treatment parameters differed substantially, including variations in therapeutic modalities (e.g., traditional acupuncture vs. moxibustion, manual vs. electroacupuncture–excluding sham or placebo acupuncture controls), acupoint selection protocols, and electroacupuncture stimulation frequencies Network meta-analyses, scoping reviews, and overviews of systematic reviews.

### 2.3 Search strategy

A comprehensive search was conducted across eight databases: Wanfang, China National Knowledge Infrastructure (CNKI), China Biology Medicine Disc (CBM), China Science and Technology Journal Database (VIP), Web of Science (WOS), PubMed, and the Cochrane Library. SR/MAs about acupuncture for PFP were systematically retrieved, with a cutoff date of June 5, 2025, for database inclusion. No language restrictions were imposed during the search process. The search strategy incorporated a combination of controlled vocabulary (MeSH/Emtree terms) and free keywords, encompassing the following terms: “acupuncture,” “electroacupuncture,” “moxibustion,” “scalp acupuncture,” “facial paralysis,” “peripheral facial palsy,” “lower motor neuron facial palsy,” “facial neuritis,” “idiopathic facial palsy,” “Bell's palsy,” “Ramsay Hunt syndrome,” “Hunt's syndrome,” “meta-analysis,” and “systematic review.” The complete search syntax for each database is provided in Supplementary Table 1.

### 2.4 Literature screening and data extraction

Two independent investigators conducted literature screening and data extraction in a blinded manner, followed by cross-verification. In cases of disagreement, a third reviewer was consulted for adjudication. Missing data were supplemented by contacting the original authors whenever feasible. Following duplicate removal using specialized software, preliminary screening was performed based on titles and abstracts. After excluding manifestly irrelevant studies, full-text articles were thoroughly reviewed to determine final eligibility for inclusion. The extracted data encompassed: (1) Basic characteristics: article title, study design, first author, publication year, total sample size, quality assessment tools, intervention protocols, and comparator groups; (2) Outcome measures assessed in the studies, including relative effects (with 95% confidence intervals), *P*-values, and primary conclusions; (3) Critical components of quality assessment: specifically, items from AMSTAR-2, GRADE evidence quality assessment tools, and PRISMA checklists.

### 2.5 Quality assessment methods for systematic reviews

The methodology, quality of reporting, and quality of evidence of the included studies were evaluated using the AMSTAR2, PRISMA statement, and GRADE tools, and each article was independently evaluated and cross-checked by two investigators, with a third evaluator invited to assist in the rating when there was disagreement.

#### 2.5.1 Inclusion of research methodological evaluations

The methodological quality of the included studies was rigorously assessed utilizing the 2017 updated and revised AMSTAR2 (17) instrument, with the 2nd, 4th, 7th, 9th, 11th, 13th, and 15th items designated as critical domains. Each area is scored as “yes”, “no” or “partially yesm” or “not applicable”. Studies were classified as follows: high quality if they fulfilled all but one or fewer non-critical domains; medium quality if they failed to meet more than one non-critical domain; low quality if they did not satisfy one critical domain; and very low quality if they were unable to meet multiple critical domains.

#### 2.5.2 Evaluation of the quality of reports included in the study

The PRISMA statement (18) was used to evaluate the quality of the reports of the included SRs/MAs, containing 27 entries, each with three scoring criteria: complete report (1 point), partial report (0.5 points), no report (0 points), and no applicable. The quality of reports was quantitatively assessed using a percentage-based scoring system, where the sum of individual item scores was divided by the theoretical maximum score (adjusted to exclude non-applicable items) and subsequently multiplied by 100. Based on the transformed scores, report quality was qualitatively categorized as follows: scores below 60 were classified as low quality, scores between 60 and 80 as moderate quality, and scores above 80 as high quality (19).

#### 2.5.3 Evaluation of the quality of evidence from included studies

The GRADE tool was used to evaluate the quality of evidence included in the SRs/MAs (20). High-quality evidence was considered if there was no downgraded evidence, moderate-quality evidence if there was one downgrade, low-quality evidence if there were two downgrades, and very-low-quality evidence if there were three or more downgrades.

### 2.6 Statistical analysis

The rapid publication of a large number of SRs/MAs for the same area of research in a short period may lead to a significant overlap of included RCTs. Such overlapping inclusion may introduce bias to the pooled results. The Corrected Covered Area (CCA) metric is conventionally employed to quantify the degree of study overlap across multiple systematic reviews (21). Our analysis utilized the Graphical Overview of Evidence (GROOVE) framework (22) to construct evidence matrices and compute CCA values. The derived CCA values were categorized according to established thresholds: 0%−5% denoting slight overlap, 6%−10% representing moderate overlap, 11%−15% indicating high overlap, and exceeding 15% signifying very high overlap. The Evidence Mapping Tool (https://www.pymeta.com/evdmap/) was employed to construct an evidence map for outcome measures. The x-axis denotes the quality of evidence, while the y-axis represents the outcome indicators. The size of each bubble is proportional to the sample size, with blue bubbles indicating statistically significant differences and yellow bubbles denoting non-significant findings.

## 3 Results

### 3.1 Literature screening process and results

**Figure 1:**
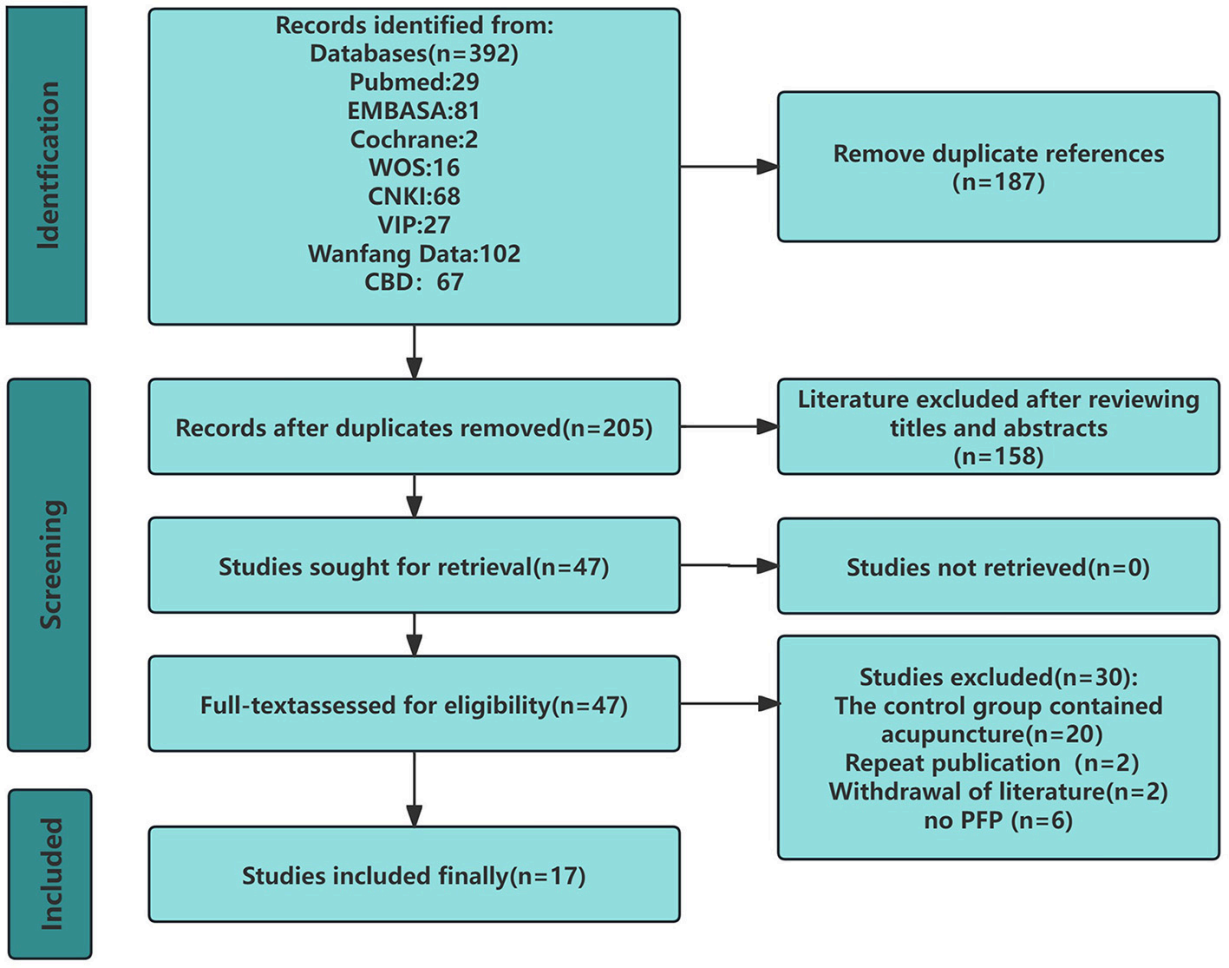
PRISMA flowchart for a comprehensive review of systematic reviews and meta-analyses of acupuncture for peripheral facial palsy.

The preliminary literature search identified a total of 392 records. Following the removal of 187 duplicate entries, 205 records remained for initial screening. Subsequent evaluation of titles and abstracts resulted in the exclusion of 158 records, leaving 47 for full-text assessment. After meticulous review, 30 articles were excluded due to non-compliance with the predefined inclusion criteria, culminating in the final inclusion of 17 studies. The comprehensive screening procedure is delineated in Figure 1.

### 3.2 Basic characteristics of the included studies

This study incorporated a total of 17 SRs and MAs (23–39) spanning the period from 2005 to 2024. Thirteen SRs and MAs exclusively incorporated RCTs, while four studies included either RCTs or CCTs. The number of included studies ranged from 3 to 27, with sample sizes varying between 200 and 2,500 participants. Among these, six were published in English and eleven in Chinese. For quality assessment, fourteen SRs and MAs utilized the Cochrane risk-of-bias tool, whereas three employed the Jadad scale. Regarding disease subtypes, 10 SRs and MAs focused on Bell's palsy, five examined peripheral facial palsy, one investigated pediatric peripheral facial palsy, and one explored idiopathic facial palsy. In terms of disease stages, five SRs and MAs addressed the acute phase, while one focused on the sequelae stage. Thirteen SRs and MAs (23–34, 37) reported positive outcomes regarding the efficacy of acupuncture for PFP, whereas four (35, 36, 38, 39) concluded that current evidence was insufficient to support its therapeutic effectiveness. The basic characteristics of all included studies are shown in Table 1.

**Table 1:** Basic characteristics of included studies.

**Inclusion of studies**	**Publication year (searching duration)**	**Type of disease**	**Type of included studies**	**Trials (subjects)**	**Treatment intervention**	**Control intervention**	**Outcomes**	**Quality assessment**	**Main conclusions**
Han (23)	2024 (inception-2022.1.21)	PFP (acute phase)	RCT, CCT	8 (896)	ACT	WMD	AE, HB, HT, AER	ROB	Acupuncture is effective in treating acute-phase peripheral facial paralysis, but high-quality evidence is needed.
Shaoying (24)	2022 (inception-2022.3.14)	PFP (post-mortem period)	RCT	10 (613)	ACT	OT	OE, HB, BPS, AER	ROB, GRADE	Acupuncture has shown efficacy in treating peripheral facial paralysis during the postictal phase; however, high-quality evidence and precise, uniform evaluation metrics are lacking.
Xue (25)	2020 (inception-2020.8.6)	Acute idiopathic facial nerve palsy	RCT	27 (2,370)	ACT	WMD	CR, OE, HT, HB	ROB, GRADE	The available evidence suggests that acute or full superficial stabbing may improve the overall clinical benefit and clinical cure of idiopathic facial nerve palsy, as well as shorten the time to symptom improvement and improve facial nerve function. It may be a potential treatment for idiopathic facial nerve palsy.
Linfeng (26)	2019 (inception– 2019.2)	PFP (children)	RCT	13 (1,196)	AT	WMD	OE, CR, FDIP	ROB	Available evidence suggests that acupuncture therapy is efficacious in the treatment of peripheral facial paralysis in children and has a strong effect on improving clinical symptoms and quality of life.
Zhang (27)	2019 (2014–2019)	PFP	RCT/CCT	27 (2,199)	AT+OT	WM, SAT, Placebo	OE, AER	ROB	Acupuncture treatment for peripheral facial paralysis is superior to conventional Western medicine.
Qian (28)	2015 (inception– 2014.3.15)	PFP	RCT	11 (915)	AT	WM, CM	OE	Jadad	The overall effective rate of pure acupuncture in the treatment of peripheral facial paralysis was significantly better than that of drug therapy, indicating its definite clinical efficacy.
Lipan (29)	2015 (inception– 2014.10)	Bell's palsy	RCT	6 (238)	AT, AT+WMD	WMD	OE, CR, AE	Jadad	Acupuncture alone healed better than WM alone. Acupuncture and moxibustion, when combined with Western medicine, were more effective than monotherapy; however, there was no significant difference in healing between acupuncture and Western medicine alone.
Lu (30)	2012 (inception– 2012)	Bell's palsy (acute phase)	RCT	17 (1,564)	AT, AT+WM	WM	CR, OE, AER	ROB	Acupuncture, as a safe and effective treatment, is suitable for intervention in the acute phase of Bell's palsy, and its clinical efficacy can be improved with conventional therapy. Western medical treatment. However, the quality of the included studies is not high, and more high-quality randomized controlled trials are needed to confirm the effectiveness of AT further.
Jiang (31)	2011 (2003–2008)	PFP acute phase	RCT	8 (933)	ACT	OT	AE	Jadad	The available clinical evidence suggests that the application of acupuncture treatment in the acute phase of peripheral facial paralysis improves the patient's symptoms and shortens the recovery time of facial paralysis by enhancing the treatment's efficacy more than it would without acupuncture treatment.
Lina (32)	2010 (1979–2009.6.30)	Bell's palsy (acute phase)	RCT	4 (316)	AT	WM, CM	OR, HT, AER	ROB	There is no difference in the cure rate of acupuncture over hormonal treatment of Bell's palsy in the acute phase. Still, there may be a trend toward a shorter course of treatment, which needs to be further demonstrated by high-quality evidence.
Li (33)	2005 (inception– 2002.12)	Bell's palsy	RCT/CCT	3 (288)	ACT	OT	Number with sequelae after 6 months, AER	ROB, AER	Acupuncture is effective in treating Bell's palsy; however, the small sample size and poor quality of the three randomized controlled trials that were enrolled lowered the reliability of this finding.
Zhang (34)	2019 (inception– 2018.7)	Bell's palsy	RCT	11 (1,258)	AT	WM	OE, CR, AER	ROB	Despite insufficient evidence of its safety, acupuncture appears to be an effective treatment for Bell's palsy.
Zhang (35)	2018 (inception– 2016.07)	Bell's palsy	RCT	20 (2,511)	AT	OT	OE, CR, AER	ROB	Due to the limited methodological quality of these studies and potential biases, it cannot be concluded that acupuncture is effective in treating facial paralysis.
Li (36)	2015 (inception– 2014.07)	Bell's palsy	RCT	14 (1,541)	ACT	OT	OE, AER	ROB	The available evidence is insufficient to support acupuncture as an effective treatment for Bell's palsy, and there is insufficient evidence to support the efficacy and safety of AT.
Kim (37)	2012 (inception– 2010.06)	Bell's palsy	RCT	8 (585)	AT, AT+WM	WM	OE, AER	ROB	Acupuncture is beneficial for the symptomatic treatment of Bell's palsy; however, the evidence is limited.
Chen (38)	2010 (inception– 2010.05.24)	Bell's palsy	RCT	6 (537)	ACT	OT	Number with sequelae after 6 months, AER	ROB	Conclusions from existing trials comparing acupuncture with no acupuncture or with other drugs are unreliable.
Zhou (39)	2009 (inception– 2006.04)	Bell's palsy	RCT/CCT	6 (537)	ACT	OT	Number with sequelae after 6 months, AER, OE	ROB	The quality of the included trials was insufficient to draw any conclusions about the efficacy of acupuncture; therefore, research from higher-quality trials is needed.

PFP, peripheral facial paralysis; RCT, randomized controlled study; CCT, controlled clinical study; ACT, acupuncture combination therapy, with acupuncture as the main intervention, may be combined with other therapies such as Western medicine; AT, acupuncture therapy alone; WMD, Western medicine drug; AE, apparent efficiency; HB, House-Brackmann facial nerve grading system; HT, healing time; AER, adverse event rate; BPS, BELL's paralysis decalogue score; OE, overall efficiency; CR, cure rate; EA, electroacupuncture; OT, other therapies after excluding acupuncture; ROB, the cochrane risk of bias assessment tool; FDIP, facial disability index physical function.

### 3.3 Overlap between included reviews (CCA)

**Figure 2:**
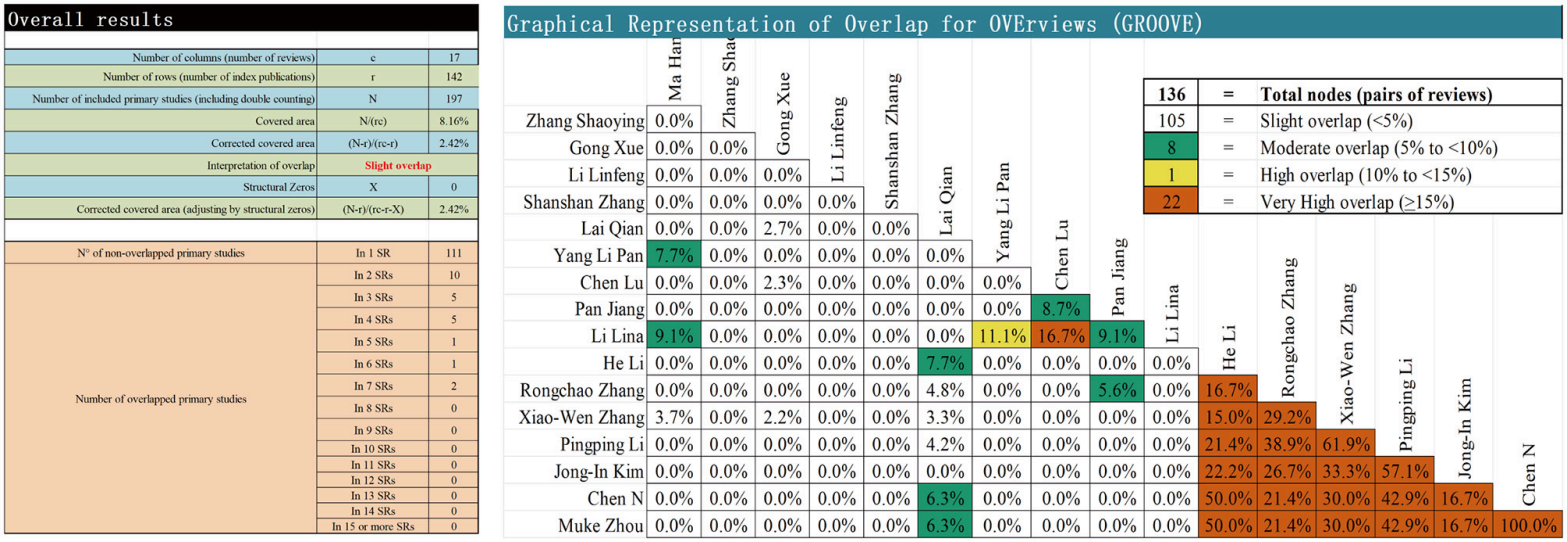
Matrix diagram of GROOVE. Overall results demonstrate the components of the CCA formula, the calculation of CCA, the calculation of CCA adjusted for structural zeros, and the absolute number of overlapping and non-overlapping primary studies.

GROOVE not only calculates the overall CCA but also provides the overlap between each pair of SRs and MAs. As shown in Figure 2, the overall CCA value for this study was 2.42% with a slight overlap. A total of 136 nodes were formed, with 22 of them exhibiting high overlap. Notably, the highly overlapping nodes were almost centered in the English study.

### 3.4 Quality evaluation of methodologies

**Figure 3:**
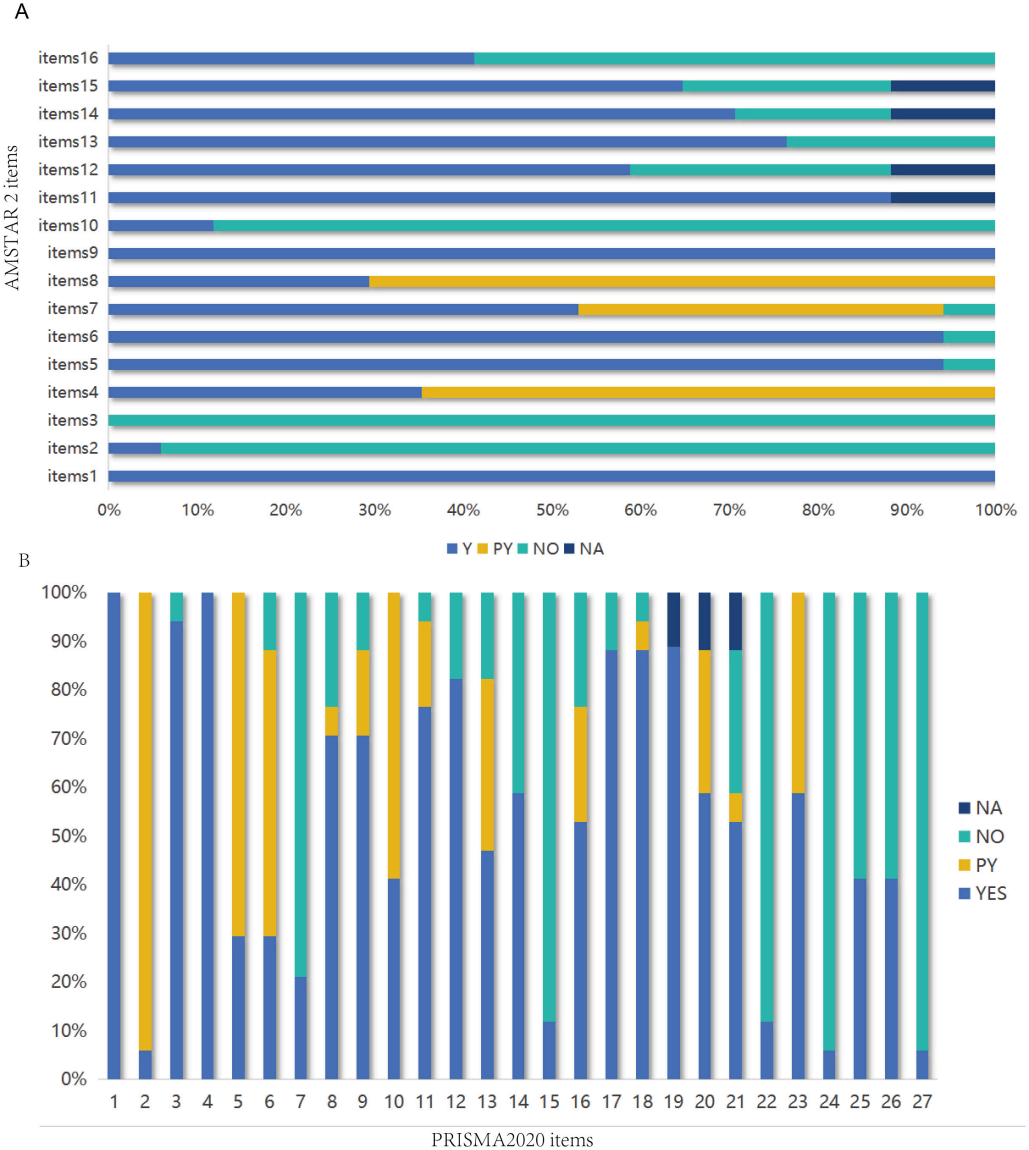
**(A)** Evaluation of methodological quality of inclusion in SRS using AMSTAR2. **(B)** Evaluation of the quality of reports included in the SRS using PRISMA2020.

Figure 3A summarizes the results of using AMSTAR 2 to assess the methodological quality of the included studies. The methodological quality of three studies (34, 36, 38) was rated as low, while fourteen (23–33, 35, 37, 39) studies were deemed to have very low methodological quality. Only items one and nine achieved a completion rate of 100%. The completion rates for items 2, 3, 4, 8, 10, and 16 were all below 50%. Registration details (Item 2) were not reported in 16 studies (23–37, 39). None of the studies provided a rationale for the inclusion criteria of the research types (Item 3). Eleven studies (23–32, 35) failed to employ a comprehensive search strategy (Item 4). Twelve studies (23–32, 36, 37) failed to provide comprehensive descriptions of the fundamental characteristics of included studies (Item 8). Fifteen studies (23–33, 38, 39) omitted disclosure of funding sources (Item 10), while 10 studies (23–28, 30–33) did not report potential conflicts of interest (Item 16). Detailed quality assessment of all included studies is presented in Supplementary Table 1.

### 3.5 Report quality evaluation

The reporting completeness of the 17 included systematic reviews (SRS) was assessed using the PRISMA checklist (Figure 3B). The results showed that the overall report quality was poor, with only two (34, 38) studies being of high quality, nine (23–26, 33, 35–37, 39) studies being of moderate quality, and six studies (19, 27–32) being of low quality. Notably, only Item 1 (Title) achieved 100% compliance across all studies. Furthermore, fewer than 50% of the SRS fully reported the following PRISMA items: Item 2 (Structured Abstract), Item 6 (Information Sources), Item 7 (Complete Search Strategy), Item 10 (Data Items), Item 13 (Synthesis Methods), Item 15 (Risk of Bias Assessment Methods), Item 22 (Risk of Bias Results), Item 24 (Protocol Registration), Item 25 (Funding), Item 26 (Conflicts of Interest), and Item 27 (Availability of Data). The report quality assessment of the included studies is shown in Supplementary Table 2.

### 3.6 Evaluation of the quality of reporting

**Figure 4:**
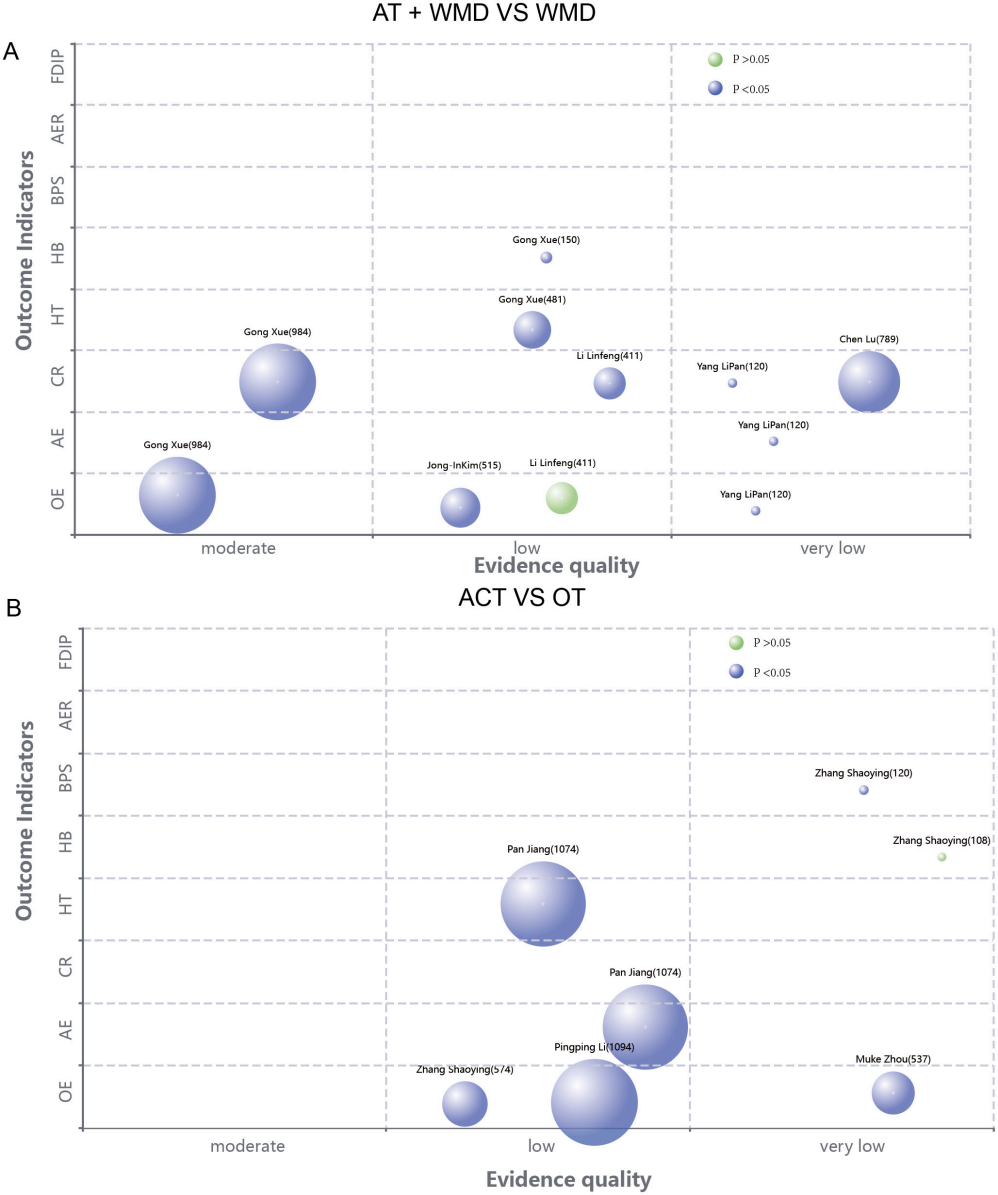
**(A)** Evidence map of the efficacy of acupuncture combined with Western medicine treatment compared with Western medicine treatment alone. **(B)** Evidence mapping of acupuncture combination therapy to other therapy outcome indicators.

**Figure 5:**
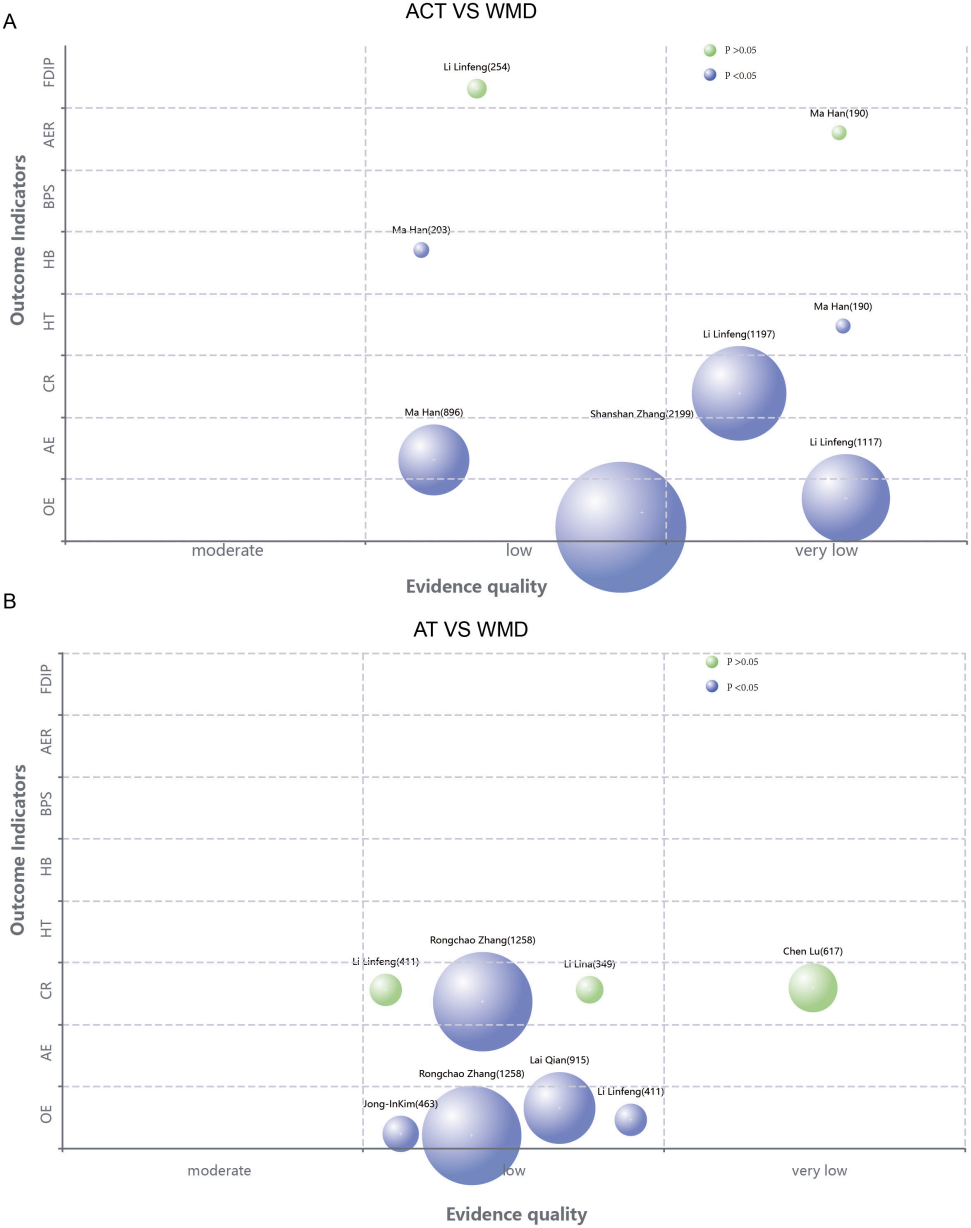
**(A)** Evidence chart of the therapeutic effect of acupuncture combined therapy and Western medicine alone. **(B)** Evidence mapping of the efficacy of acupuncture compared with other therapies.

**Figure 6:**
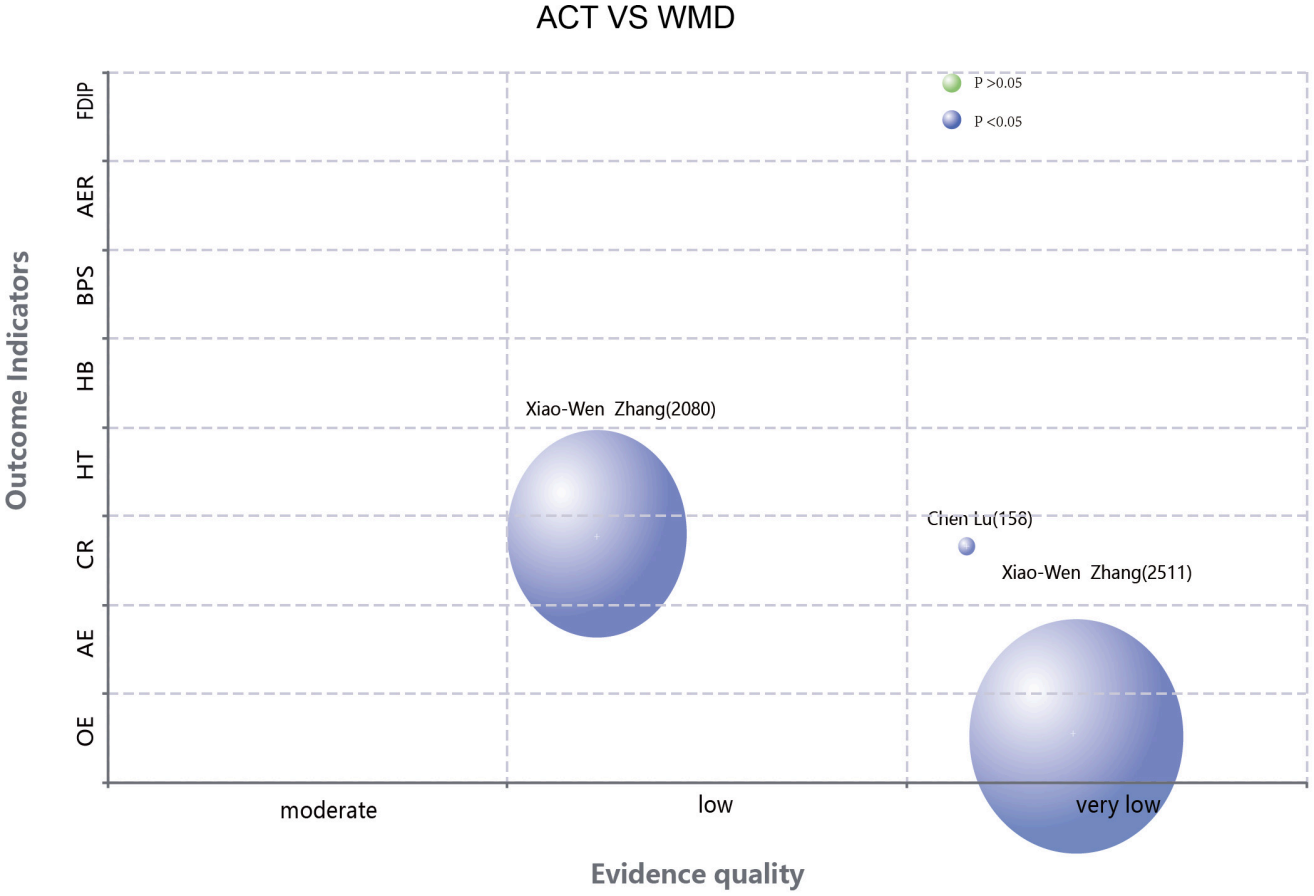
Evidence mapping of the efficacy of acupuncture compared with other therapies.

A total of 39 outcome-related items from 17 SRS reports were evaluated using the GRADE system (Figures 4–6). The results demonstrated that the majority of the findings were supported by low-quality evidence, with no high-quality evidence identified. Only two items (5.12%) were classified as moderate-quality evidence, while 22 items (56.41%) were rated as low-quality evidence, and an additional 13 items (33.33%) were deemed to be of very low-quality evidence. Risk of bias constitutes the most prevalent factor contributing to downgrading, followed by inconsistency, imprecision, and publication bias. Table 2 presents the pertinent outcome measures along with comprehensive details about the GRADE evaluation results.

**Table 2:** Quality of evidence from included studies.

**Inclusion of studies**	**Interventions (treatment group vs. control group)**	**Outcome (number of studies)**	**Efficiency value**	**95% CI**	***P*-value**	**Risk of bias**	**Inconsistency**	**Indirectness**	**Inaccuracy**	**Publication bias**	**Level of evidence**
Han (23)	ACT vs. WM	AE (8,896)	OR = 2.36	1.43, 3.90	0.0008	−1^a^	0	0	0	−1^f^	Low
		HB (2,203)	WMD = −0.27	−0.49, −0.06	0.01	−1^a^	0	0	−1^e^	0	Low
		HT (2,190)	WMD = −0.63	−0.93, −0.34	<0.0001	−2^b^	0	0	−1^e^	0	Very low
		AER (2,190)	OR = 0.27	0.05, 1.34	0.11	−2^b^	0	0	−1^e^	0	Very low
Shaoying (24)	ACT vs. OT	OE (9,574)	OR = 3.26	2.12, 5.02	<0.0001	−1^a^	0	0	0	−1^f^	Low
		HB (2,108)	WMD = −0.77	−1.90, 0.36	0.18	−1^a^	0	0	−1^e^	−1^f^	Very low
		BPS (2,120)	WMD = −2.33	−3.25,−1.42	<0.0001	−1^a^	0	0	−1^e^	−1^f^	Very low
		AER (2)	NA	NA		NA	NA	NA	NA	NA	NA
Xue (25)	AT+WMD vs. WMD	OE (10,984)	OR = 5.52	3.19–9.55	<0.001	−1^a^	0	0	0	0	Moderate
		CR (10,984)	OR = 3.35	2.53–4.45	<0.001	−1^a^	0	0	0	0	Moderate
		HT (4,481)	SMD = 1.81	2.03–1.6	<0.00001	−1^a^	0	0	−1^e^	0	Low
		HB (2,150)	SMD = 0.64	0.97–0.32	0.0001	−1^a^	0	0	−1^e^	0	Low
Linfeng (26)	ACT vs. WMD	OE (121,117)	RR = 1.22	1.16–1.28	<0.001	−1^a^	−1^c^	0	0	−1^f^	Very low
		CR (131,197)	RR = 1.89	1.49–2.41	<0.001	−1^a^	−1^c^	0	0	−1^f^	Very low
		FDIP (3,254)	RR = 3.76	−10.83 to 18.34	0.61	−1^a^	−1	0	−1^e^	0	Low
	AT vs. WMD	OE (3,411)	RR = 1.14	1.03–1.26	0.0005	−1^a^	0	0	−1^e^	0	Low
		CR (3,411)	RR = 1.91	0.91–4.02	0.09	−1^a^	0	0	−1^e^	0	Low
	AT+WMD vs. WMD	OE (152)	RR = 1.02	0.91–1.16	0.71	−1^a^	0	0	−1^e^	0	Low
		CR (2,132)	RR = 1.28	1.10–1.50	0.002	−1^a^	0	0	−1^e^	0	Low
Zhang (27)	ACT vs. WMD	OE (272,199)	OR = 6.30	4.60–8.63	<0.001	−1^a^	0	0	0	−1^f^	Low
	PFP	AER	NA	NA							
Qian (28)	AT vs. WMD	OE (11,915)	OR = 5.46	3.24–9.22	<0.001	−1^a^	0	0	0	−1^f^	Low
LiPan (29)	AT+WMD vs. WMD	OE (2,120)	OR = 4.36	1.44–13.20	0.009	−1^a^	0	0	−1^e^	−1^f^	Very low
		CR (2,120)	OR = 4.36	1.44–13.20	0.009	−1^a^	0	0	−1^e^	−1^f^	Very low
		AE (2,120)	OR = 4.36	1.44–13.20	0.09	1^a^	0	0	−1^e^	−1^f^	Very low
Lu (30)	AT vs. WM	CR (7,617)	RR = 1.16	0.93, 1.45	0.20	−2^b^	−1	0	0	−1^e^	Very low
	AT+WM vs. WM	CR (8,789)	RR = 1.25	1.08, 1.46	0.004	−2^b^	−1	0	0	−1^e^	Very low
	AT vs. TDP	CR (2,158)	RR = 1.67	1.21, 2.31	0.002	−2^b^	0	0	−1^d^	0	Very low
Jiang (31)	ACT vs. OT PFP	AE (81,074)	OR = 3.27	2.20, 4.87	<0.0001	−1^a^	0	0	0	−1^e^	Low
		HT (81,074)	WMD = −3.86	−4.44, 3.27	<0.0001	−1^a^	0	0	0	−1^e^	Low
Lina (32)	AT vs. WMD	CR (4,349)	RR = 1.14	0.88–1.48	0.33	−1^a^	0	0	−1^d^	0	Low
		HT (3)	NA	NA	NA	–	–	–	–	–	
Li (33)	ACT vs. OT	OE	NA	NA	NA	–	–	–	–	–	
Zhang (34)	AT vs. WMD	CR (111,258)	RR = 1.77	1.41–2.21	0.00008	−1^a^	−1^c^	0	0	0	Low
		OE (111,258)	RR = 1.18	1.07–1.31	0.001	−1^a^	−1^c^	0	0	0	Low
Zhang (35)	AT vs. OT	OE (202,511)	RR = 1.11	1.05–1.17	<0.005	−1^a^	−1^c^	0	0	−1^e^	Very low
		CR (152,080)	RR = 1.56	1.30–1.87	<0.0001	−1^a^	−1^c^	0	0	0	Low
Li (36)	ACT vs. OT	OE (131,094)	RR = 1.14	1.04–1.25	0.005	−1^a^	−1^c^	0	0	0	Low
	AT+WM vs. WM	OE (8)	RR = 1.14	1.08, 1.20	<0.0001	−1^a^	0	0	0	0	Moderate
	AT vs. OT	OE (7)	1.11	1.00, 1.24	0.05	−1^a^	−1^c^	0	0	0	Low
	AT vs. WMD	OE (6)	1.18	1.02, 1.36	0.02	−1^a^	−1^c^	0	0	0	Low
Kim (37)	AT vs. WMD	OE (4,463)	RR = 1.07	1.02–1.13	0.006	−2^b^	0	0	0	0	Low
	AT+WMD vs. WMD	OE (6,515)	RR = 1.11	1.05–1.17	0.0001	−2^b^	0	0	0	0	Low
Chen (38)	ACT vs. OT	NA	NA	NA	NA	–	–	–	–	–	–
Zhou (39)	ACT vs. OT	OE (6.537)	RR = 1.33	1.21–1.47	<0.0001	−1^a^	−1^c^	0	0	−1^e^	Very low

^a^ Most of the included studies had a moderate risk of bias, downgraded by one level.

^b^ Most of the included studies had a high risk of bias, downgraded by two levels.

^c^ The point estimate was too large, the confidence intervals did not overlap, the heterogeneity P value was too small, and the I^2^ was too large.

^d^ The sample size was too small or the total confidence interval was too wide.

^e^ Publication bias existed.

### 3.7 Efficacy evaluation

#### 3.7.1 The overall efficacy rates

A total of 12 SRS studies synthesized the overall efficacy rates of acupuncture or acupuncture combined with other therapies for PFP. Meta-analyses from 11 SRS demonstrated that acupuncture interventions exhibited significantly higher overall efficacy rates compared to non-acupuncture controls (*P* <0.05) (24–29, 34–37, 39). One SRS (33) conducted a qualitative analysis of three included randomized controlled trials (RCTs), consistently showing superior efficacy of acupuncture over control groups in PFP. Regarding disease stages, one SRS reported that acupuncture yielded significantly better outcomes (OR = 3.26, 95% CI: 2.12–5.02) than other therapies during the sequela phase of PFP (24). Another SRS found acupuncture more efficacious (OR = 5.52, 95% CI: 3.19–9.55) for acute-stage PFP compared to other treatments (25). Subgroup analyses of intervention modalities revealed that four SRS identified standalone acupuncture as statistically superior to pharmacotherapy (*P* <0.05) (26, 28, 34, 37). Additionally, five SRS demonstrated that combined acupuncture and pharmacotherapy achieved better therapeutic outcomes than pharmacotherapy alone (*P* <0.05) (25, 26, 29, 36, 37). However, a study shows that the total effective rate of acupuncture combined with Western medicine in treatment is not superior to that of using Western medicine alone (RR = 1.02, 95% CI: 0.91–1.16) (26). A study showed that acupuncture combined therapy was superior to Western medicine alone in treating PFP in children (RR = 1.22, 95% CI: 1.16–1.28) (26).

#### 3.7.2 The cure rate and healing time

Seven (25, 26, 29, 30, 32, 34, 35) SRs were conducted to evaluate the cure rate of acupuncture in treating PFP. The results demonstrated that acupuncture, either as a standalone intervention or in combination with other therapeutic modalities, significantly enhanced the cure rate of PFP compared to non-acupuncture treatments (*P* <0.05). In terms of disease staging, three studies demonstrated significantly better recovery rates with acupuncture therapy for acute-phase PFP than with other therapies (25, 30, 32). Subgroup analyses showed that the cure rate of acupuncture alone was higher than that of Western medicine alone (25, 34). However, three studies (26, 30, 32) found no statistically significant difference between the acupuncture and Western medicine groups. In addition, acupuncture alone had a better recovery rate than TDP treatment (30). Four studies showed that the healing time was significantly shorter when acupuncture was used either alone or in combination with other therapies compared to no acupuncture (*P* <0.05) (23, 25, 31, 32).

#### 3.7.3 The apparent efficiency

Three SRS (23, 29, 31) studies showed that acupuncture was more effective than other therapies in treating PFP and demonstrated positive effects in the acute phase.

#### 3.7.4 Facial paralysis motor function evaluation scale

Three (23, 24, 31) studies employed the HB as an outcome measure. Among these, two (23, 31) studies demonstrated that the acupuncture group exhibited superior improvement in HB scores compared to the control group. However, one (24) study found no statistically significant advantage of acupuncture over the control group (WMD: −0.77, 95% CI: −1.90 to 0.36, *P* = 0.18). One study (24) used BPS as an outcome indicator and showed that acupuncture was superior to other therapies in improving BPS scores in the acute phase of PFP. An SRS (26) evaluated FDIP values and showed that there was no statistically significant difference between the acupuncture group and the Western medicine group (RR=3.76, 95% CI: −10.83 to 18.34).

#### 3.7.5 Adverse event rate

Twelve SRSs reported adverse events in the included studies (23–25, 27, 30, 32, 33, 35–39), of which eight SRSs reported no reported or no adverse reactions in their included studies (24, 30, 32–34, 36–39). One SRS reported no statistically significant difference in the incidence of adverse reactions in the acupuncture group vs. the control group (23). Three SRS reported manifestations of needle-sickness, such as panic, dry mouth, and bitter mouth in the acupuncture group, which were improved by drinking water with no residual symptoms (25, 27, 35).

## 4 Discussion

### 4.1 A summary of quality assessment for included studies

Acupuncture, as a physical therapy with the advantages of simplicity, low side effects, and low economic cost, has been widely used in the treatment of PFP. Accompanying the increase in clinical studies related to the treatment of PFP with acupuncture in recent years, several systematic reviews have emerged to assess the quality of the efficacy of acupuncture in the treatment of PFP. Although systematic reviews are located at the top of the clinical evidence pyramid, only quality evaluations generated by correct systematic reviews using methods consistent with evidence-based medicine are reliable. This study summarizes the quality of 17 SRS of acupuncture for PFP. Notably, the majority of RCTs were conducted in Chinese populations. This phenomenon may be attributed to China's well-established acupuncture treatment system and clinical practices, which facilitate large-scale clinical studies by researchers. A bibliometric analysis from 2013 to 2023 showed that China contributed over 50% of the research in the field of acupuncture for treating facial paralysis (14). However, this geographic concentration raises concerns about the generalizability of our findings to other ethnic groups and healthcare settings. There is an urgent need for high-quality multinational RCTs to confirm the benefits of acupuncture for peripheral facial paralysis across different ethnic populations and establish its global clinical relevance.

Excessive overlap among the included studies in the SRS may compromise the precision of the results, potentially introducing bias in the outcomes of certain interventions (40, 41). In the present study, the CCA indicated a slight overall degree of overlap among the incorporated SRs, suggesting that, on average, these reviews draw upon largely non-redundant bodies of evidence. Nevertheless, we note that several pairwise comparisons exhibited substantial overlap—such as between Zhang (35) and Li (36) (61.9%), Chen (38) and Zhou (39) (100%)—highlighting the risk of redundant evidence being interpreted as independent corroboration. This could mislead clinicians and overstate the robustness of certain intervention effects. Therefore, while our synthesis adds value, it also underscores the need for greater transparency and pre-registration to avoid unnecessary duplication.

Most of these studies suffered from methodological flaws and incomplete reporting. The AMSTAR-2 assessment revealed several prevalent methodological shortcomings in the reviewed studies: (1) failure to register study protocols *a priori*, (2) inadequate justification for the inclusion of specific study designs, (3) incomplete literature search strategies, (4) insufficient reporting of baseline characteristics of included studies, and (5) non-disclosure of funding sources for incorporated research. Additionally, Chinese-language publications frequently exhibited two critical deficiencies: failure to account for potential bias risk in overall effect estimates and non-disclosure of conflicts of interest.

First, the lack of pre-registration may lead to selective reporting bias, as researchers might be more inclined to include papers with positive results. Second, due to the diversity of outcome measures for PFP, researchers may only report outcome measures that show positive results after conducting a meta-analysis. Additionally, as mentioned above, pre-registration helps to avoid duplication of efforts. To enhance methodological rigor, researchers should prospectively register systematic reviews with either Cochrane or PROSPERO before commencement. Any protocol deviations occurring during the research process should be explicitly documented and justified, as such practices promote research transparency and mitigate potential biases (42). The AMSTAR2 guidelines require researchers to provide a rationale for the selection of study types included in their systematic reviews. Current studies predominantly specify the inclusion of RCTs without elucidating the underlying justification. Although RCTs are methodologically rigorous compared to other study designs, when confronted with limitations such as insufficient RCT literature or highly restricted study populations, researchers should either explicitly address how these constraints impact their findings or consider broadening the scope of eligible study types to achieve more comprehensive results (18). Most existing studies fail to provide detailed search strategies, which not only affects publication bias but also reduces the reproducibility of the research. Most importantly, we cannot confirm whether the search strategies used in SRs are comprehensive, covering all subtypes of PFP and various types of acupuncture interventions. Comprehensive literature retrieval serves as a pivotal methodology to ensure the exhaustiveness of results and mitigate publication bias, wherein researchers should prioritize both database searches and the acquisition of gray literature. An elaborate delineation of included studies facilitates subsequent researchers in ascertaining the conformity of selected literature with PICO criteria and detecting potential heterogeneity. In instances where included studies exhibit varying risks of bias, researchers ought to conduct subgroup analyses to elucidate potential sources of bias. Systematic reviewers are obligated to explicitly disclose funding sources and potential conflicts of interest, thereby enabling readers to evaluate possible biases in the review outcomes.

These deficiencies are also evident in the reporting quality assessed by PRISMA. Additionally, the included studies exhibit further reporting shortcomings, such as insufficiently detailed abstracts, absence of methodologies and results for evaluating evidence credibility (thereby casting doubt on the reliability of the findings), failure to contextualize the discussion within the broader evidence base (which hinders subsequent researchers' efforts to synthesize existing evidence), and lack of accessibility to research data, code, and Supplementary materials (thereby compromising transparency).

The publication date of the studies included in the analysis may be one reason for their lower quality. AMSTAR-2 was published in 2017, PRISMA 2020 in 2020, whereas most of the SRS included in this study were published before 2020. The immaturity of the methodology may be another reason for their lower quality. In summary, the prevalent methodological limitations in current SRs/MAs collectively indicate that the body of evidence regarding the efficacy of acupuncture may be subject to significant bias risks. Potential biases—including selective reporting, publication bias, and undisclosed conflicts—tend to skew toward overestimating treatment effects. Therefore, optimistic conclusions derived from many reviewed syntheses should be interpreted with considerable caution. Future systematic reviews not only need to adhere to rigorous methodological standards but also explicitly discuss how adherence to these standards can prevent inflated effect estimates, thereby providing more reliable and unbiased assessments of acupuncture.

### 4.2 The efficacy and evidence credibility of acupuncture in treating PFP

The majority of studies have demonstrated the positive effects of acupuncture in treating PFP. The findings of this study indicate that the overall response rate serves as a key outcome measure in relevant systematic reviews. Notably, both acupuncture monotherapy and acupuncture combined with other therapeutic modalities exhibit superior overall response rates compared to non-acupuncture interventions. The relevant systematic reviews categorize various types of acupuncture therapies under the general term “acupuncture.” However, the type of acupuncture is a factor influencing treatment efficacy. Grouping them together may overestimate or underestimate the effectiveness of specific acupuncture types. Therefore, conducting subgroup analyses or network meta-analyses to parse out the heterogeneity caused by different acupuncture types would be beneficial for guiding clinical practice. However, no studies have yet systematically analyzed this issue. Acupuncture intervention has demonstrated enhanced therapeutic efficacy across various subtypes, stages, and demographic groups of PFP. Bell's palsy represents the most prevalent subtype of PFP, which is consistent with the findings of the present study, wherein 58.8% of the SRS were focused on Bell's palsy. Ramsay Hunt syndrome represents the second most prevalent subtype of PFP, characterized by ipsilateral peripheral facial nerve paralysis induced by varicella-zoster virus infection, typically accompanied by severe otalgia, herpetiform eruptions, and potential auditory or vestibular dysfunction (43). Although no systematic reviews on acupuncture treatment for Hunter syndrome have been conducted to date, several case reports have demonstrated its therapeutic potential (44–46).

Currently, the management of the acute phase of PFP predominantly relies on pharmacological interventions, while the application of acupuncture during this phase remains contentious, with divergent expert opinions regarding its therapeutic efficacy and optimal timing (3). The results of this study showed that either acupuncture combined with other therapies or acupuncture therapy alone improved the overall effectiveness of treating PFP in the acute phase. However, these studies did not have the same criteria for determining the acute phase, with three SRSs (23, 25, 32) clearly defining the acute phase as occurring within 7 days of onset, and two SRSs (31, 32) no explicitly defining the acute phase. There remains considerable debate regarding whether the acute phase of PFP should be defined as 7 or 15 days, which has introduced heterogeneity in the interpretation of research findings and potentially compromised their reliability (47).

The duration of recovery is a critical concern for patients, as it affects facial muscles. In terms of improving their healing rates, various studies have yielded different results, which may be attributed to the sample size of the included studies and the inconsistency of the evaluation criteria for healing used in the original studies. The use of multiple outcome measures complicates the synthesis of data. Although standardized mean differences (SMDs) enable statistical pooling across different scales, they do not eliminate the challenges associated with clinical interpretability. Statistically significant SMDs may not translate into clinically meaningful improvements in facial symmetry or function, especially when the original scales differ in sensitivity and reliability. Currently, the most commonly used evaluation and efficacy criteria for peripheral facial nerve palsy include H-B evaluation criteria, the Sunnybrook (Toronto) score, FDIP, the therapeutic criteria for symptoms of facial paralysis (facial neuritis) in traditional Chinese medicine, and the evaluation of facial nerve function. There are differences in the assessment of healing between these scales (48–50). The H-B and Toronto Facial Grading Systems are the two most widely utilized clinical scales for facial nerve assessment. However, both exhibit notable limitations, including relatively imprecise evaluation criteria for normal facial nerve function, failure to incorporate complications such as crocodile tears, contractures, and spasms, and susceptibility to inter-rater variability in subjective assessments. Six outcome measures based on facial nerve function scores were included in this study; four (23–25) indicated that acupuncture therapy was superior to other therapies, and two (24, 26) indicated that acupuncture therapy was not statistically significant compared to other therapies. This variability in conclusions may stem from differences in evaluation metrics. The development of artificial intelligence (AI) may offer a solution to this issue, as multiple algorithms have already been applied to the assessment and prognosis evaluation of PFP (51–53).

Abnormalities caused by PFP exert detrimental effects on self-esteem, confidence, and psychological wellbeing (54). Consequently, therapeutic interventions aimed at minimizing recovery time are of paramount importance. Most patients recovered within 3 months after treatment, and the use of acupuncture therapy significantly shortened the healing time compared to Western medicine alone. However, a subset of patients continues to exhibit residual symptoms beyond the 3-month onset period, accompanied by manifestations such as facial muscle tension, spasms, and synkinesis on the affected side (55). At this stage, there remains a paucity of well-established and universally recognized therapeutic interventions. Clinically, intermittent administration of neurotrophic agents is predominantly employed as a palliative measure (56). The present study incorporated an SRS, which demonstrated the efficacy of acupuncture in treating the sequelae stage of PFP, with concomitant improvements in facial nerve functionality.

Although PFP occurs predominantly in adults in their 20s and 40s, the treatment of peripheral facial palsy in children presents greater challenges (57). Compared to adults, pediatric patients with idiopathic facial nerve palsy exhibit superior spontaneous recovery capacity; however, a substantial proportion still experience delayed recovery, imposing significant psychological burdens on their families (58). Given the uncertain therapeutic benefits of corticosteroids and parental concerns regarding potential adverse effects (59), there is an urgent need for complementary and alternative therapeutic approaches. A systematic review and meta-analysis encompassing 13 RCTs involving 1,196 pediatric patients with peripheral facial paralysis demonstrated that acupuncture significantly enhanced both the overall response rate and complete recovery rate, while exhibiting substantial efficacy in ameliorating clinical symptoms and improving quality of life (26).

Our study shows the effectiveness of acupuncture in different populations and stages of facial paralysis. However, it is essential to note that the efficacy of acupuncture exhibits somatic specificity and acupoint specificity, which are influenced by factors such as the distribution of nerve fibers at specific sites, the intensity, and depth of needle insertion (60). This suggests that different needling techniques and practitioners may impact the therapeutic outcomes. It is essential to conduct subsequent multi-center, large-sample real-world studies to analyze the actual effects of acupuncture in clinical practice and explore its influencing factors. Therefore, it is necessary to conduct multi-center, large-sample, real-world studies to analyze the actual impact of acupuncture in clinical practice and explore its influencing factors. Additionally, for pediatric PFP patients, adherence is a key concern. Fear of acupuncture may reduce adherence. Although the SRs included in this study did not address adherence, previous studies have shown that acupuncture is feasible and acceptable for children (61).

## 5 Conclusion

In summary, although our study results suggest that acupuncture therapy has positive effects on improving clinical outcomes, enhancing facial nerve function, shortening recovery periods, and enhancing the quality of life for patients with facial paralysis, while also demonstrating good safety. This therapeutic modality applies to diverse PFP subtypes, disease stages, and patient populations. However, methodological limitations in existing SRs and their included primary studies substantially compromise the reliability of the evidence. Future research should prioritize rigorously designed RCTs to validate the therapeutic efficacy and safety of acupuncture for PFP. Such high-quality evidence will be instrumental in upgrading evidence hierarchies, thereby facilitating evidence-based clinical decision-making and the development of guidelines.

## 6 Advantages and limitations

This study possesses several notable strengths. Firstly, a comprehensive search was conducted across eight Chinese and English databases to systematically collect systematic reviews and meta-analyses about acupuncture and combined acupuncture therapies for PFP, thereby summarizing the efficacy and safety of these treatments. Secondly, the methodological and reporting quality of the included meta-analyses was rigorously evaluated using the AMSTAR-2 and PRISMA 2020 guidelines. Thirdly, the quality of the synthesized evidence was assessed via the GRADE approach. Fourthly, the quality of evidence and effect sizes for each outcome measure were visually represented using bubble plots. Lastly, the degree of overlap among the included primary studies was quantified using the GROOVE tool.

This study has several limitations. Primarily, the number of included studies was limited by small sample sizes. Additionally, substantial heterogeneity was observed among the included studies. It is generally accepted that evidence is considered reliable only when it reaches at least moderate quality (62). The risk of bias constitutes a primary factor compromising the quality of evidence, with the majority of primary studies included in the SRS demonstrating moderate-to-high risk profiles. This phenomenon stems from several methodological limitations: inadequate reporting or improper implementation of randomization procedures, predisposing to selection bias; inherent challenges in blinding due to the distinctive nature of acupuncture interventions; and substantial heterogeneity in therapeutic protocols encompassing needling techniques, timing of intervention, acupoint selection, manipulation methods, stimulation intensity, and treatment duration–all contributing to both elevated bias risk and clinical inconsistency. Subsequent researchers conducting relevant RCTs should refer to the CONSORT (63) extension statement for acupuncture RCTs and employ evidence-based methodologies to scientifically design randomization procedures, blinding protocols, and acupuncture-specific details, thereby facilitating the conduct of multicenter, large-scale, high-quality RCTs.

Restricting inclusion to Chinese- and English-language systematic reviews may introduce publication bias. Many Chinese SRs on acupuncture for facial palsy lack rigorous methodology—such as risk of bias assessment or publication bias evaluation—potentially inflating effect estimates. Moreover, relevant reviews from Japan and Korea, where acupuncture is commonly practiced, may be published in local languages and thus overlooked. As an overview, the exclusion of non-Chinese/non-English literature may limit the representativeness of the evidence.

## Data Availability

The original contributions presented in the study are included in the article/Supplementary material, further inquiries can be directed to the corresponding author.

## References

[1] Singh A Deshmukh P. Bell's palsy: a review. Cureus. (2022) 14:e30186. doi: 10.7759/cureus.30186 36397921 PMC9648613

[2] Rajangam J Lakshmanan AP Rao KU Jayashree D Radhakrishnan R Roshitha B . Bell palsy: facts and current research perspectives. Cns Neurol Disord Drug Targets. (2024) 23:203–14. doi: 10.2174/1871527322666230321120618 36959147

[3] Baugh RF Basura GJ Ishii LE Schwartz SR Drumheller CM Burkholder R . Clinical practice guideline: bell's palsy executive summary. Otolaryngol Head Neck Surg. (2013) 149:656–63. doi: 10.1177/0194599813506835 24190889

[4] De Seta D Mancini P Minni A Prosperini L De Seta E Attanasio G . Bell's palsy: symptoms preceding and accompanying the facial paresis. ScientificWorldJournal. (2014) 2014:801971. doi: 10.1155/2014/801971 25544960 PMC4270115

[5] Butler DP Morales DR Johnson K Nduka C. Facial palsy: when and why to refer for specialist care. Br J Gen Pract. (2019) 69:579–80. doi: 10.3399/bjgp19X706541 31672833 PMC6808583

[6] Peitersen E. Bell's palsy: the spontaneous course of 2,500 peripheral facial nerve palsies of different etiologies. Acta Otolaryngol Suppl. (2002):4–30. doi: 10.1080/000164802320401694 12482166

[7] Luu NN Chorath KT May BR Bhuiyan N Moreira AG Rajasekaran K. Clinical practice guidelines in idiopathic facial paralysis: systematic review using the appraisal of guidelines for research and evaluation (agree II) instrument. J Neurol. (2021) 268:1847–56. doi: 10.1007/s00415-020-10345-0 33389026

[8] Deng X Zhu H Shi L Li Y Shi H Wu Y . Comparison of the efficacy of acupuncture with tuina with acupuncture-only in the treatment of peripheral facial paralysis: a network meta-analysis. Intern Emerg Med. (2024) 19:839–58. doi: 10.1007/s11739-024-03562-2 38483737 PMC11039505

[9] Shan Z. Electron microscope observation of acupuncture and nerve repair in the treatment of peripheral facial paralysis. Emerg Med Int. (2022) 2022:5432223. doi: 10.1155/2022/5432223 35875246 PMC9300289

[10] Huang YL Yu W Zhang L Chen HB. A controlled study on the therapeutic effect of acupuncture and acupuncture combined with drugs on peripheral facial paralysis with normal result of facial nervemagnetic resonance examination. Zhongguo Zhen Jiu. (2019) 39:139–42. 30942031 10.13703/j.0255-2930.2019.02.007

[11] The 64 indications for acupuncture recognised by the World Health Organisation. Chin Acup. (2008) 28(S1):65.

[12] Duan W Chen D Huang Z Zeng Y Liu S Wang C . Biological effect of acupuncture on peripheral facial paralysis. Front Neurol. (2025) 16:1516904. doi: 10.3389/fneur.2025.1516904 40352774 PMC12063603

[13] Liu ZH Qi YC Pan PG Ma MM Qian XG Fu WJ . Effect of acupuncture-moxibustion combined with nerve growth factor on compensation of cerebral function in the children of cerebral palsy. Zhongguo Zhen Jiu. (2007) 27:565–8. 17853751

[14] Lan D Huang C Yu N Lao J Li Z. Research trends of acupuncture therapy on facial paralysis in a decade spanning 2013-2023: a bibliometric analysis. Complement Ther Med. (2023) 79:103006. doi: 10.1016/j.ctim.2023.103006 37972694

[15] Pollock M Fernandes RM Becker LA Featherstone R Hartling L. What guidance is available for researchers conducting overviews of reviews of healthcare interventions? A scoping review and qualitative metasummary. Syst Rev. (2016) 5:190. doi: 10.1186/s13643-016-0367-5 27842604 PMC5109841

[16] Miao RQ Zhu FY Wang TY Yin S Shuai C Li T . The effectiveness and safety of acupuncture for post-stroke depression: an overview of systematic reviews. Complement Ther Med. (2025) 91:103178. doi: 10.1016/j.ctim.2025.103178 40258536

[17] Shea BJ Reeves BC Wells G Thuku M Hamel C Moran J . AMSTAR 2: a critical appraisal tool for systematic reviews that include randomised or non-randomised studies of healthcare interventions, or both. BMJ. (2017) 358:j4008. doi: 10.1136/bmj.j4008 28935701 PMC5833365

[18] Page MJ Moher D Bossuyt PM Boutron I Hoffmann TC Mulrow CD . PRISMA 2020 explanation and elaboration: updated guidance and exemplars for reporting systematic reviews. BMJ. (2021) 372:n160. doi: 10.1136/bmj.n160 33781993 PMC8005925

[19] Chen R Zhou X Deng G Li S Li L. Assessment of quality of reporting and methodology in systematic reviews of moxibustion for chronic diseases using PRISMA 2020 and AMSTAR 2. Complement Ther Med. (2025) 92:103193. doi: 10.1016/j.ctim.2025.103193 40381744

[20] Pollock A Farmer SE Brady MC Langhorne P Mead GE Mehrholz J . An algorithm was developed to assign grade levels of evidence to comparisons within systematic reviews. J Clin Epidemiol. (2016) 70:106–10. doi: 10.1016/j.jclinepi.2015.08.013 26341023 PMC4742519

[21] Pieper D Antoine SL Mathes T Neugebauer EA Eikermann M. Systematic review finds overlapping reviews were not mentioned in every other overview. J Clin Epidemiol. (2014) 67:368–75. doi: 10.1016/j.jclinepi.2013.11.007 24581293

[22] Perez-Bracchiglione J Meza N Bangdiwala SI Nino DGE Urrutia G Bonfill X . Graphical representation of overlap for overviews: groove tool. Res Synth Methods. (2022) 13:381–8. doi: 10.1002/jrsm.1557 35278030

[23] Han M Yongqiang P Xi Z Yanlong X. Meta-analysis of outcome indicators of acupuncture combined therapy versus hormone therapy for acute peripheral facial nerve palsy. Chin Ethn Folk Med. (2024) 33:100–6. doi: 10.3969/j.issn.1007-8517.2024.09.zgmzmjyyzz202409022

[24] Shaoying Z Jianfang Z Yidan C Pengfei Q. Meta-analysis of the efficacy of acupuncture in treating peripheral facial paralysis in the sequelae period. Zhejiang Clin Med. (2022) 24:1335–8.

[25] Xue G Xiaotian Z Yu W Jun Z Yi L. Evaluation of the efficacy and safety of shallow acupuncture in the treatment of acute idiopathic facial nerve palsy. J Zunyi Med Univ. (2020) 43:764–78.

[26] Linfeng L Baoqiang D Mingxing M Guangming L Xingxing L. Meta-analysis of the clinical efficacy of acupuncture therapy in the treatment of peripheral facial paralysis in children. J Tradit Chin Med Clin Pract. (2019) 31:1660–6.

[27] Shanshan Z Guohui L Xinrong G Zhipeng L Xixi D. Meta-analysis of acupuncture for peripheral facial paralysis. New Chin Med. (2019) 51:204–8.

[28] Qian L Fangyuan Z Zhenjie Y. A systematic review of the efficacy of acupuncture alone versus drug therapy for peripheral facial paralysis. West Tradit Chin Med. (2015) 28:59–62.

[29] Lipan Y Yan L Jinghui Z. Systematic review and meta-analysis of the efficacy of acupuncture combined with Western medicine in the treatment of Bell's palsy. J Pract Tradit Chin Med. (2015) 29:4–7.

[30] Lu C Suhuo L Xiaoyi Z. A systematic review of the efficacy and safety of acupuncture treatment for acute Bell's palsy. J Tradit Chin Med. (2012) 53:1921–6.

[31] Jiang P Wei Z Wusheng C Wenying S. A systematic review of acupuncture treatment for peripheral facial paralysis (acute phase). Clin J Acupunct Med Moxibustion. (2011) 27:60–3. doi: 10.3969/j.issn.1005-0779.2011.04.029

[32] Lina L Bo L Jun X Yuanhao D. A systematic review of the efficacy of acupuncture versus hormone therapy in the acute phase of Bell's palsy. J Liaoning Univ Tradit Chin Med. (2010) 12:97–9.

[33] He L Zhou M Zhou D Li N Wu B. A systematic review of the efficacy of acupuncture in the treatment of Bell's palsy. Chin J Evid-Based Med. (2005) 106–9.

[34] Zhang R Wu T Wang R Wang D Liu Q. Compare the efficacy of acupuncture with drugs in the treatment of bell's palsy: a systematic review and meta-analysis of RCTs. Medicine. (2019) 98:e15566. doi: 10.1097/MD.0000000000015566 31083225 PMC6531040

[35] Zhang XW Wang FM Yu SS Zhou QH. The effect of acupuncture on bell's palsy: an overall and cumulative meta-analysis of randomized controlled trials. Int J Clin Exp Med. (2018) 11:3309–21.

[36] Li P Qiu T Qin C. Efficacy of acupuncture for bell's palsy: a systematic review and meta-analysis of randomized controlled trials. PLoS ONE. (2015) 10:e121880. doi: 10.1371/journal.pone.0121880 25974022 PMC4431843

[37] Kim JI Lee MS Choi TY Lee H Kwon HJ. Acupuncture for bell's palsy: a systematic review and meta-analysis. Chin J Integr Med. (2012) 18:48–55. doi: 10.1007/s11655-011-0861-5 21994030

[38] Chen N Zhou M He L Zhou D Li N. Acupuncture for bell's palsy. Cochrane Database Syst Rev. (2010) 2010:CD2914. doi: 10.1002/14651858.CD002914.pub5 PMC7133542 20687071

[39] Zhou M He L Zhou D Wu B Li N Kong S . Acupuncture for bell's palsy. J Altern Complement Med. (2009) 15:759–64. doi: 10.1089/acm.2008.0179 19500005

[40] Chen SY Zhang XR Chen J Ge WQ Wang WW Wang XS . An overview of meta-analyses of endovascular bridging therapies for acute ischemic stroke. Biomed Res Int. (2018) 2018:9831210. doi: 10.1155/2018/9831210 29707581 PMC5863300

[41] Ioannidis JP. The mass production of redundant, misleading, and conflicted systematic reviews and meta-analyses. Milbank Q. (2016) 94:485–514. doi: 10.1111/1468-0009.12210 27620683 PMC5020151

[42] Tawfik GM Giang H Ghozy S Altibi AM Kandil H Le HH . Protocol registration issues of systematic review and meta-analysis studies: a survey of global researchers. Bmc Med Res Methodol. (2020) 20:213. doi: 10.1186/s12874-020-01094-9 32842968 PMC7448304

[43] Goswami Y Gaurkar SS. Ramsay hunt syndrome: an introduction, signs and symptoms, and treatment. Cureus. (2023) 15:e33688. doi: 10.7759/cureus.33688 36793818 PMC9925029

[44] Giralt SI Carvajal G Garcia-Janeras A Faba A Nishishinya AM. A severe case of Ramsay hunt syndrome treated with acupuncture and related techniques. Complement Ther Clin Pract. (2020) 39:101119. doi: 10.1016/j.ctcp.2020.101119 32379658

[45] Zheng RW Liu D Eric TE Ning YZ Chen LL Hu H . A case study of Ramsay hunt syndrome in conjunction with cranial polyneuritis. Medicine. (2017) 96:e8833. doi: 10.1097/MD.0000000000008833 29381990 PMC5708989

[46] Li J Wang J Li G Zhang J Zhang B Wang S. Severe postherpetic neuralgia and facial paralysis in the oral and periauricular regions managed with acupuncture and electroacupuncture: a case report. Front Pain Res. (2024) 5:1474103. doi: 10.3389/fpain.2024.1474103 39763628 PMC11701028

[47] Chen S Li Y Mo X Yan P Hu X Sun C . Reflections and suggestions on the researches of acupuncture-moxibustion for idiopathic facial palsy. Zhongguo Zhen Jiu. (2025) 45:379–84. 40097224 10.13703/j.0255-2930.20240110-0002

[48] Jeong J Lee JM Cho YS Kim J. Inter-rater discrepancy of the House-Brackmann facial nerve grading system. Clin Otolaryngol. (2022) 47:680–3. doi: 10.1111/coa.13956 35818896

[49] Gonzalez-Cardero E Infante-Cossio P Cayuela A Acosta-Feria M Gutierrez-Perez JL. Facial disability index (FDI): adaptation to Spanish, reliability and validity. Med Oral Patol Oral Cir Bucal. (2012) 17:e1006–12. doi: 10.4317/medoral.18054 22926474 PMC3505694

[50] VanSwearingen JM Brach JS. The facial disability index: reliability and validity of a disability assessment instrument for disorders of the facial neuromuscular system. Phys Ther. (1996) 76:1288–98, 1298–300. doi: 10.1093/ptj/76.12.1288 8959998

[51] Zhang Y Gao W Yu H Dong J Xia Y. Artificial intelligence-based facial palsy evaluation: a survey. IEEE Trans Neural Syst Rehabil Eng. (2024) 32:3116–34. doi: 10.1109/TNSRE.2024.3447881 39172615

[52] Boochoon K Mottaghi A Aziz A Pepper JP. Deep learning for the assessment of facial nerve palsy: opportunities and challenges. Facial Plast Surg. (2023) 39:508–11. doi: 10.1055/s-0043-1769805 37290452

[53] Lee SA Kim J Lee JM Hong YJ Kim IJ Lee JD. Automatic facial recognition system assisted-facial asymmetry scale using facial landmarks. Otol Neurotol. (2020) 41:1140–8. doi: 10.1097/MAO.0000000000002735 33169952

[54] Huang B Xu S Xiong J Huang G Zhang M Wang W. Psychological factors are closely associated with the bell's palsy: a case-control study. J Huazhong Univ Sci Technolog Med Sci. (2012) 32:272–9. doi: 10.1007/s11596-012-0048-0 22528233

[55] Snyder V Frost AS Ciolek PJ. Advances in facial reanimation. Otolaryngol Clin North Am. (2023) 56:599–609. doi: 10.1016/j.otc.2023.02.020 37003859

[56] Heckmann JG Urban PP Pitz S Guntinas-Lichius O Gagyor I. The diagnosis and treatment of idiopathic facial paresis (bell's palsy). Dtsch Arztebl Int. (2019) 116:692–702. doi: 10.3238/arztebl.2019.0692 31709978 PMC6865187

[57] de Almeida JR Guyatt GH Sud S Dorion J Hill MD Kolber MR . Management of bell palsy: clinical practice guideline. CMAJ. (2014) 186:917–22. doi: 10.1503/cmaj.131801 24934895 PMC4150706

[58] Babl FE Mackay M Dalziel SR. Facial nerve palsy in children. J Paediatr Child Health. (2019) 55:878–9. doi: 10.1111/jpc.14500 31270864

[59] Salman MS MacGregor DL. Should children with bell's palsy be treated with corticosteroids? A systematic review. J Child Neurol. (2001) 16:565–8. doi: 10.1177/088307380101600805 11510926

[60] Liu S Wang Z Su Y Qi L Yang W Fu M . A neuroanatomical basis for electroacupuncture to drive the vagal-adrenal axis. Nature. (2021) 598:641–5. doi: 10.1038/s41586-021-04001-4 34646018 PMC9178665

[61] Brittner M Le Pertel N Gold MA. Acupuncture in pediatrics. Curr Probl Pediatr Adolesc Health Care. (2016) 46:179–83. doi: 10.1016/j.cppeds.2015.12.005 26867822

[62] Guyatt GH Oxman AD Vist GE Kunz R Falck-Ytter Y Alonso-Coello P . Grade: an emerging consensus on rating quality of evidence and strength of recommendations. BMJ. (2008) 336:924–6. doi: 10.1136/bmj.39489.470347.AD 18436948 PMC2335261

[63] MacPherson H Altman DG Hammerschlag R Youping L Taixiang W White A . Revised standards for reporting interventions in clinical trials of acupuncture (stricta): extending the consort statement. J Altern Complement Med. (2010) 16:ST1–14. doi: 10.1089/acm.2010.1610 20954957

