# Supplementary material for: The efficacy and safety of acupuncture treatment for peripheral facial paralysis: an overview of systematic review and meta-analysis
Source: Front Neurol. 2025 Nov 11;16:1669551. doi: 10.3389/fneur.2025.1669551 (PMC12644010; doi:10.3389/fneur.2025.1669551)
Supplement: Supplementary file 1 [file Table_1.doc]

Detailed search strategy for each database

**PUBMED**

("Facial Paralysis"[Mesh] OR "Bell Palsy"[Mesh] OR "Ramsay Hunt Syndrome"[Mesh] OR "Facial Nerve Diseases"[Mesh]

OR "facial paralysis"[tiab] OR "facial palsy"[tiab] OR "peripheral facial paralysis"[tiab] OR "Bell's palsy"[tiab]

OR "idiopathic facial palsy"[tiab] OR "facial neuritis"[tiab] OR "Ramsay Hunt"[tiab] OR "Hunt's syndrome"[tiab]

OR "upper motor neuron facial palsy"[tiab])

AND

("Acupuncture Therapy"[Mesh] OR "Acupuncture Points"[Mesh] OR "Electroacupuncture"[Mesh] OR "Moxibustion"[Mesh]

OR "Acupuncture"[tiab] OR "acupuncture therapy"[tiab] OR "electroacupuncture"[tiab] OR "moxibustion"[tiab]

OR "acupoint"[tiab] OR "acupuncture point"[tiab] OR "auricular acupuncture"[tiab] OR "ear acupuncture"[tiab]

OR "pharmacoacupuncture"[tiab] OR "acupotomy"[tiab] OR "pharmacopuncture"[tiab])

AND

("Meta-Analysis as Topic"[Mesh] OR "Systematic Reviews as Topic"[Mesh] OR "Review Literature as Topic"[Mesh]

OR "meta-analysis"[pt] OR "systematic review"[pt]

OR "meta analysis"[tiab] OR "metaanalysis"[tiab] OR "systematic review"[tiab] OR "systematic literature review"[tiab])

**WOS**

TS=("Acupuncture Treatment" OR "Acupuncture Treatments" OR "Treatment, Acupuncture" OR "Therapy, Acupuncture" OR "Pharmacoacupuncture Treatment" OR "Pharmacoacupuncture Treatments" OR "Treatment, Pharmacoacupuncture" OR "Therapy, Pharmacoacupuncture" OR Acupotomy OR Acupotomies OR "Acupunctures, Ear" OR "Ear Acupunctures" OR "Acupuncture, Auricular" OR "Acupunctures, Auricular" OR "Auricular Acupunctures" OR "Auricular Acupuncture" OR "Ear Acupuncture" OR "Acupuncture Point" OR "Point, Acupuncture" OR "Points, Acupuncture" OR "Acupoints" OR "Acupoint" OR "Electroacupuncture" OR "Electro-acupuncture" OR "Electroacupuncture Therapy" OR "Electro-acupuncture Therapy" OR "Scalp Acupuncture" OR "Scalp Acupunctures" OR "Scalp Needling" OR "Cephalic Acupuncture")

AND TS=("Facial Paralysis" OR "Paralyses, Facial" OR "Paralysis, Facial" OR "Facial Palsy" OR "Facial Palsies" OR "Palsies, Facial" OR "Palsy, Facial" OR "Hemifacial Paralysis" OR "Paralyses, Hemifacial" OR "Paralysis, Hemifacial" OR "Facial Palsy, Lower Motor Neuron" OR "Facial Paralysis, Peripheral" OR "Facial Paralyses, Peripheral" OR "Paralysis, Peripheral Facial" OR "Peripheral Facial Paralysis" OR "Lower Motor Neuron Facial Palsy" OR "Facial Paresis" OR "Pareses, Facial" OR "Paresis, Facials" OR "Bell's Palsy" OR "Bell Palsy" OR "Idiopathic Facial Paralysis" OR "Hunt Syndrome" OR "Ramsay Hunt Syndrome" OR "Herpes Zoster Oticus" OR "Facial Palsy, Ramsay Hunt") AND TS=("systematic review" OR "systematic reviews as topic" OR "meta-analysis" OR "meta-analyses")

**EMBASE**

('facial paralysis'/exp OR 'bell palsy'/exp OR 'ramsay hunt syndrome'/exp OR 'facial nerve disease'/exp OR 'facial paralysis':ti,ab OR 'facial palsy':ti,ab OR 'peripheral facial paralysis':ti,ab OR 'bell's palsy':ti,ab OR 'idiopathic facial palsy':ti,ab OR 'facial neuritis':ti,ab OR 'ramsay hunt':ti,ab OR 'hunt's syndrome':ti,ab OR 'upper motor neuron facial palsy':ti,ab) AND ('acupuncture'/exp OR 'electroacupuncture'/exp OR 'moxibustion'/exp OR 'acupuncture point'/exp OR 'acupuncture':ti,ab OR 'acupuncture therapy':ti,ab OR 'electroacupuncture':ti,ab OR 'moxibustion':ti,ab OR 'acupoint':ti,ab OR 'auricular acupuncture':ti,ab OR 'ear acupuncture':ti,ab OR 'pharmacoacupuncture':ti,ab OR 'acupotomy':ti,ab OR 'pharmacopuncture':ti,ab) AND ('meta analysis'/exp OR 'systematic review'/exp OR 'meta analysis':pt OR 'systematic review':pt OR 'meta analysis':ti,ab OR 'metaanalysis':ti,ab OR 'systematic review':ti,ab OR 'systematic literature review':ti,ab)

**Cochrane Library**

#1 (acupuncture OR "acupuncture therapy" OR "ear acupuncture" OR "acupuncture points" OR electroacupuncture OR "scalp acupuncture" OR pharmacoacupuncture) IN Title, Abstract, Keywords

#2 MeSH descriptor: [Acupuncture] explode all trees

#3 MeSH descriptor: [Acupuncture Points] explode all trees

#4 MeSH descriptor: [Acupuncture Therapy] explode all trees

#5 MeSH descriptor: [Acupuncture, Ear] explode all trees

#6 MeSH descriptor: [Acupuncture, Ear] explode all trees

#7 MeSH descriptor: [Electroacupuncture] explode all trees

#8 #1 OR #2 OR #3 OR #4 OR #5 OR #6 OR #7

#9 MeSH descriptor: [Bell Palsy] explode all trees

#10 MeSH descriptor: [Facial Paralysis] explode all trees

#11 "Peripheral Facial Paralysis":ti,ab,kw OR "Peripheral Facial Palsy":ti,ab,kw OR "Bell Palsy":ti,ab,kw OR "Bell's Palsy":ti,ab,kw OR "Idiopathic Facial Paralysis":ti,ab,kw OR "Idiopathic Facial Palsy":ti,ab,kw OR "Facial Nerve Palsy":ti,ab,kw OR "Facial Nerve Paralysis":ti,ab,kw OR "Seventh Nerve Palsy":ti,ab,kw OR "Seventh Nerve Paralysis":ti,ab,kw OR "Ramsay Hunt Syndrome":ti,ab,kw

#12 #9 OR #10 OR #11

#13 MeSH descriptor: [Meta-Analysis as Topic] explode all trees

#14 MeSH descriptor: [Systematic Reviews as Topic] explode all trees

#15 systematic review

#16 meta-analysis

#17 #13 OR #14 OR #15 OR #16

#18 #8 AND #12 AND #17

**中国知网**

SU=('面神经炎' OR '周围性面瘫' OR '周围性面神经麻痹' OR '贝尔面瘫' OR 'Hunt综合征' OR '特发性面神经麻痹')

AND

SU=('针刺' OR '电针' OR '头针' OR '艾灸' OR '针灸' OR '温针' OR '耳针' OR '热敏灸' OR '耳穴')

AND

SU=('系统评价' OR 'Meta分析' OR '荟萃分析' OR '元分析')

**维普网**

(M=('面神经炎' OR '周围性面瘫' OR '周围性面神经麻痹' OR '贝尔面瘫' OR 'Hunt综合征' OR '特发性面神经麻痹')

AND

M=('针刺' OR '电针' OR '头针' OR '艾灸' OR '针灸' OR '温针' OR '耳针' OR '热敏灸' OR '耳穴')

AND

T=('系统评价' OR 'Meta分析' OR '荟萃分析' OR '元分析'))

**万方**

(题名或关键词:("面神经炎" OR "周围性面瘫" OR "周围性面神经麻痹" OR "贝尔面瘫" OR "Hunt综合征" OR "特发性面神经麻痹"))

AND

(题名或关键词:("针刺" OR "电针" OR "头针" OR "艾灸" OR "针灸" OR "温针" OR "耳针" OR "热敏灸" OR "耳穴"))

AND

(题名或关键词:("系统评价" OR "Meta分析" OR "荟萃分析" OR "元分析"))

**中国生物医学数据库**

( "面神经炎"[常用字段:智能] OR "周围性面瘫"[常用字段:智能] OR "周围性面神经麻痹"[常用字段:智能] OR "贝尔面瘫"[常用字段:智能] OR "Hunt综合征"[常用字段:智能] OR "特发性面神经麻痹"[常用字段:智能]) AND( "针刺"[常用字段:智能] OR "电针"[常用字段:智能] OR "头针"[常用字段:智能] OR "艾灸"[常用字段:智能] OR "针灸"[常用字段:智能] OR "温针"[常用字段:智能] OR "耳针"[常用字段:智能] OR "热敏灸"[常用字段:智能] OR "耳穴"[常用字段:智能]) AND( "系统评价"[常用字段:智能] OR "Meta分析"[常用字段:智能] OR "荟萃分析"[常用字段:智能] OR "元分析"[常用字段:智能])
