# Supplementary material for: The efficacy and safety of acupuncture treatment for peripheral facial paralysis: an overview of systematic review and meta-analysis
Source: Front Neurol. 2025 Nov 11;16:1669551. doi: 10.3389/fneur.2025.1669551 (PMC12644010; doi:10.3389/fneur.2025.1669551)
Supplement: Supplementary file 2 [file Table_2.doc]

**Table 1Supplementary Table 2: Assessment of methodological quality of included studies using AMSTAR 2**

| ncluded studies | ① | ②^*^ | ③ | ④^*^ | ⑤ | ⑥ | ⑦^*^ | ⑧ | ⑨^*^ | ⑩ | ⑪^*^ | ⑫ | ⑬^*^ | ⑭ | ⑮^*^ | ⑯ | quality grade |
| --- | --- | --- | --- | --- | --- | --- | --- | --- | --- | --- | --- | --- | --- | --- | --- | --- | --- |
| Ma Han | Y | N | N | PY | Y | Y | Y | PY | Y | N | Y | Y | Y | Y | Y | N | Very low |
| Zhang Shaoying | Y | N | N | PY | Y | Y | PY | PY | Y | N | Y | Y | Y | Y | N | N | Very low |
| Gong Xue | Y | N | N | PY | Y | Y | Y | PY | Y | N | Y | Y | Y | Y | Y | N | Very low |
| Li Linfeng | Y | N | N | PY | Y | Y | Y | PY | Y | N | Y | Y | Y | Y | Y | N | Very low |
| Shanshan Zhang | Y | N | N | PY | Y | Y | PY | PY | Y | N | Y | N | Y | Y | Y | N | Very low |
| Lai Qian | Y | N | N | PY | Y | Y | PY | PY | Y | N | Y | Y | Y | Y | Y | N | Very low |
| Yang Li Pan | Y | N | N | PY | Y | Y | PY | PY | Y | N | Y | N | N | N | N | Y | Very low |
| Chen Lu | Y | N | N | PY | Y | Y | PY | PY | Y | N | Y | N | N | Y | Y | N | Very low |
| Pan Jiang | Y | N | N | PY | N | N | N | PY | Y | N | Y | N | N | N | Y | N | Very low |
| Li Lina | Y | N | N | PY | Y | Y | PY | PY | Y | N | Y | N | N | N | Y | N | Very low |
| He Li | Y | N | N | Y | Y | Y | Y | Y | Y | N | NA | NA | Y | NA | NA | N | Very low |
| Rongchao Zhang | Y | N | N | Y | Y | Y | Y | Y | Y | N | Y | Y | Y | Y | Y | Y | low |
| Xiao-Wen Zhang | Y | N | N | PY | Y | Y | Y | Y | Y | N | Y | Y | Y | Y | Y | Y | Very low |
| Pingping Li | Y | N | N | Y | Y | Y | Y | PY | Y | N | Y | Y | Y | Y | Y | Y | low |
| Jong-In Kim | Y | N | N | Y | Y | Y | Y | PY | Y | N | Y | Y | Y | Y | N | Y | Very low |
| Chen N | Y | Y | N | Y | Y | Y | Y | Y | Y | Y | NA | NA | Y | NA | NA | Y | low |
| Muke Zhou | Y | N | N | Y | Y | Y | PY | Y | Y | Y | Y | Y | Y | Y | N | Y | Very low |

Note: Y: Fully compliant; PY: Partially compliant; N: Non-compliant; ①: The systematic review is based on the PICO principle; ②: A registration protocol is provided; ③: The criteria for including studies are explained; ④: A comprehensive search strategy was used; ⑤: Two people independently screened the literature; ⑥: Two people independently extracted the data; ⑦: A list of excluded studies and the reasons for exclusion are provided; ⑧: The basic characteristics of the studies are described in detail; ⑨: Used appropriate tools to assess the risk of bias in included studies; ⑩: Reported funding information for included studies in the systematic review; Item 11: Selected appropriate statistical methods for conducting the meta-analysis; Item 12: Evaluated the impact of individual study bias risk on meta-analysis results; Item 13: Considered the bias risk of included studies when interpreting and discussing the results of the systematic review; Item 14: Provided reasonable explanations and discussions if heterogeneity was present; Item 15: Assessed publication bias and its impact; Item 16: Described the funding sources and conflicts of interest of this study;

**Supplementary Table 3**: Report quality of included studies assessed using PRISMA 2020

| included studies | 1 | 2 | 3 | 4 | 5 | 6 | 7 | 8 | 9 | 10 | 11 | 12 | 13 | 14 | 15 | 16 | 17 | 18 | 19 | 20 | 21 | 22 | 23 | 24 | 25 | 26 | 27 | raw score | quality score | quality grade |
| --- | --- | --- | --- | --- | --- | --- | --- | --- | --- | --- | --- | --- | --- | --- | --- | --- | --- | --- | --- | --- | --- | --- | --- | --- | --- | --- | --- | --- | --- | --- |
| Ma Han | 1 | 0.5 | 1 | 1 | 0.5 | 0.5 | 0 | 1 | 1 | 0.5 | 1 | 1 | 0.5 | 0 | 0 | 1 | 1 | 1 | 1 | 1 | 1 | 0 | 1 | 0 | 0 | 0 | 0 | 16.5 | 61.11111111 | moderate |
| Zhang Shaoying | 1 | 0.5 | 1 | 1 | 0.5 | 0.5 | 0 | 0.5 | 0.5 | 0.5 | 1 | 1 | 0.5 | 1 | 1 | 0.5 | 1 | 1 | 1 | 1 | 1 | 1 | 0.5 | 0 | 0 | 0 | 0 | 17.5 | 64.81481481 | moderate |
| Gong Xue | 1 | 0.5 | 1 | 1 | 0.5 | 0.5 | 0 | 1 | 1 | 0.5 | 1 | 1 | 0.5 | 1 | 1 | 1 | 1 | 1 | 1 | 1 | 1 | 1 | 1 | 0 | 0 | 0 | 0 | 19.5 | 72.22222222 | moderate |
| Li Linfeng | 1 | 0.5 | 1 | 1 | 0.5 | 0.5 | 0 | 1 | 1 | 0.5 | 0.5 | 1 | 0 | 1 | 0 | 1 | 1 | 1 | 1 | 1 | 1 | 0 | 1 | 0 | 0 | 0 | 0 | 16.5 | 61.11111111 | moderate |
| Shanshan Zhang | 1 | 0.5 | 1 | 1 | 0.5 | 0.5 | 0 | 0 | 1 | 0.5 | 1 | 1 | 0.5 | 0 | 0 | 0 | 1 | 1 | 1 | 0.5 | 0.5 | 0 | 0.5 | 0 | 1 | 0 | 0 | 14 | 51.85185185 | low |
| Lai Qian | 1 | 0.5 | 1 | 1 | 0.5 | 0.5 | 0 | 1 | 1 | 0.5 | 0 | 0 | 0.5 | 0 | 0 | 0 | 1 | 0.5 | 1 | 0.5 | 1 | 0 | 0.5 | 0 | 0 | 0 | 0 | 12 | 44.44444444 | low |
| Yang Li Pan | 1 | 0.5 | 1 | 1 | 0.5 | 0.5 | 0 | 0 | 0 | 0.5 | 0.5 | 0 | 0 | 0 | 0 | 0 | 0 | 1 | 1 | 0.5 | 0 | 0 | 0.5 | 0 | 0 | 1 | 0 | 9.5 | 35.18518519 | low |
| Chen Lu | 1 | 0.5 | 1 | 1 | 0.5 | 0.5 | 0 | 0 | 0.5 | 0.5 | 1 | 0 | 0.5 | 0 | 0 | 0.5 | 1 | 1 | 1 | 1 | 1 | 0 | 1 | 0 | 0 | 0 | 0 | 13.5 | 50 | low |
| Pan Jiang | 1 | 0.5 | 1 | 1 | 0.5 | 0 | 0 | 0 | 0 | 0.5 | 0.5 | 1 | 0 | 1 | 0 | 0 | 0 | 0 | 1 | 0.5 | 1 | 0 | 0.5 | 0 | 0 | 0 | 0 | 10 | 37.03703704 | low |
| Li Lina | 1 | 0.5 | 1 | 1 | 0.5 | 1 | 0 | 1 | 0.5 | 0.5 | 1 | 1 | 1 | 0 | 0 | 0.5 | 1 | 1 | 1 | 1 | 0 | 0 | 0.5 | 0 | 0 | 0 | 0 | 15 | 55.55555556 | low |
| He Li | 1 | 0.5 | 1 | 1 | 1 | 0.5 | 0 | 1 | 1 | 1 | 1 | 1 | 1 | 1 | 0 | 1 | 1 | 1 | NA | NA | NA | 0 | 0.5 | 0 | 1 | 0 | 0 | 16.5 | 68.75 | moderate |
| Rongchao Zhang | 1 | 0.5 | 1 | 1 | 1 | 1 | 1 | 1 | 1 | 1 | 1 | 1 | 1 | 1 | 0 | 1 | 1 | 1 | 1 | 1 | 1 | 0 | 1 | 0 | 1 | 1 | 0 | 22.5 | 83.33333333 | high |
| Xiao-Wen Zhang | 1 | 0.5 | 1 | 1 | 1 | 1 | 1 | 1 | 1 | 1 | 1 | 1 | 1 | 1 | 0 | 1 | 1 | 1 | 1 | 1 | 0 | 0 | 1 | 0 | 1 | 1 | 0 | 21.5 | 79.62962963 | moderate |
| Pingping Li | 1 | 0.5 | 1 | 1 | 0.5 | 1 | 0 | 1 | 1 | 1 | 1 | 1 | 1 | 1 | 0 | 1 | 1 | 1 | 1 | 1 | 1 | 0 | 1 | 0 | 1 | 1 | 0 | 21 | 77.77777778 | moderate |
| Jong-In Kim | 1 | 0.5 | 1 | 1 | 0.5 | 1 | 1 | 1 | 1 | 1 | 1 | 1 | 1 | 0 | 0 | 1 | 1 | 1 | 1 | 1 | 0 | 0 | 1 | 0 | 1 | 1 | 0 | 20 | 74.07407407 | moderate |
| Chen N | 1 | 1 | 1 | 1 | 1 | 1 | 1 | 1 | 1 | 1 | 1 | 1 | 1 | 1 | 0 | 1 | 1 | 1 | NA | NA | NA | 0 | 1 | 1 | 0 | 1 | 1 | 21 | 87.5 | high |
| Muke Zhou | 1 | 0.5 | 1 | 1 | 1 | 0 | 0 | 1 | 1 | 1 | 1 | 1 | 1 | 1 | 0 | 0.5 | 1 | 1 | 1 | 0.5 | 0 | 0 | 1 | 0 | 1 | 1 | 0 | 18.5 | 68.51851852 | moderate |

1: Clearly state that it is a systematic review; 2: Structured abstract; 3: Theoretical basis of the study; 4: State the purpose of the study or the problem to be addressed; 5. Detail the inclusion and exclusion criteria; 6: Detail the sources of literature; 7: Complete search strategy; 8: Detail the literature screening methods; 9: Detailed description of data extraction methods; 10: Detailed description of data items; 11: Detailed description of methods for assessing risk of bias; 12: Detailed description of effect measures; 13: Detailed description of statistical methods; 14: Description of methods for assessing reporting bias; 15: Description of methods for assessing the credibility of evidence; 16: Detailed description of the literature screening process and results; 17: Report on the characteristics of included studies; 18: Report the results of bias risk assessment for included studies; 19: Report individual study results; 20: Report the synthesis of results; 21: Report publication bias; 22: Report the results of evidence credibility; 23: Conduct a comprehensive discussion of the study results; 24: Whether a registration and protocol are provided; 25: Funding support; 26: Declaration of conflicts of interest; 27: Whether complete data, code, and other materials are available.
